# Associations Between Dairy Consumption and Nutrient Intake in Southeast Asian Children: Findings from the South East Asian Nutrition Surveys II (SEANUTS II)

**DOI:** 10.3390/nu17233740

**Published:** 2025-11-28

**Authors:** Nadja Mikulic, Cécile M. Singh-Povel, Swee Ai Ng, Nga Thuy Tran, Van Khanh Tran, Rini Sekartini, Dian Novita Chandra, Bee Koon Poh, Jyh Eiin Wong, Nipa Rojroongwasinkul, Nawarat Vongvimetee, Nanda de Groot, Ilse Khouw

**Affiliations:** 1 FrieslandCampina, 3818 LE Amersfoort, The Netherlandssweeai.ng@frieslandcampina.com (S.A.N.); nanda.degroot@frieslandcampina.com (N.d.G.); ilse.tan-khouw@frieslandcampina.com (I.K.); 2 National Institute of Nutrition, Hanoi 100000, Vietnam; thuynga1997@gmail.com (N.T.T.); trankhanhvan.nin@gmail.com (V.K.T.); 3 Faculty of Medicine, Universitas Indonesia, Jakarta 10430, Indonesia; rsekartini@yahoo.com (R.S.); diannovitach@yahoo.com (D.N.C.); 4 Faculty of Health Sciences, Universiti Kebangsaan Malaysia, Kuala Lumpur 50300, Malaysia; pbkoon@ukm.edu.my (B.K.P.); wjeiin@ukm.edu.my (J.E.W.); 5 Institute of Nutrition, Mahidol University, Nakhon Pathom 73170, Thailand; nipa.roj@mahidol.ac.th (N.R.); nawarat.von@mahidol.ac.th (N.V.)

**Keywords:** dairy, nutrient intake, children, Southeast Asia

## Abstract

**Background**: Malnutrition among Southeast Asian children remains an issue. Previous studies have linked higher dairy consumption to improved nutrient intake. This study examines the impact of dairy consumption on food, energy, and nutrient intakes among 2- to 12-year-old Southeast Asian children. **Methods**: We analyzed data from the cross-sectional South East Asian Nutrition Surveys (SEANUTS) II, involving 10,299 children aged 2 to 12 years from Indonesia, Malaysia, Thailand, and Vietnam. Dietary intake was assessed using 24 h dietary recall. **Results**: Daily dairy consumption was generally low among children aged 2–12 years in Southeast Asia. The proportion meeting daily dairy recommendations was 24% in Indonesia, 17% in Malaysia, 23% in Thailand, and 8% in Vietnam. In younger children aged 2–3 years, this proportion was higher (20–55%), whereas in older children aged 7–12 years, it was lower (2.7–9.6%). In addition, high dairy intake was associated with lower consumption of extra foods, higher overall nutrient intake, and higher proportions of children meeting estimated energy requirements and recommended intakes for vitamins A, B_2_, B_12_, D, and calcium. **Conclusions**: Dairy consumption was associated with lower consumption of extra foods, and it enhanced nutrient intake among Southeast Asian children, indicating a beneficial impact on their diet.

## 1. Introduction

Ensuring adequate intake of nutrients, including protein, vitamins, and minerals, is important for children, as they are at a critical period of growth and bone mass development [1,2]. Dairy products are a rich source of many nutrients, providing energy, protein, and micronutrients such as calcium, magnesium, phosphorus, and vitamins B_2_ and B_12_ [3]. Furthermore, dairy products have been reported to be beneficial for children’s growth, particularly in enhancing bone mineral content [1,4] and reducing stunting [1,5,6]. Southeast Asia (SEA) has a relatively high prevalence of malnutrition. Among children aged 6 to 12 months, stunting has a prevalence of ~9%. Furthermore, most children in SEA fail to meet the recommended nutrient intake (RNI) for vitamin D (~96%) and calcium (~82%) [7,8,9,10]. Additionally, there is a high prevalence of vitamin D insufficiency (~26%) among those aged 4 to 12 years [7,8,9,10]. Also, dairy intake is low, only 24%, 17%, 23%, and 8% of children aged 2 to 12 years in Indonesia, Malaysia, Thailand, and Vietnam, respectively, meet the local daily dairy recommendations, with these percentage decreasing with age [11].

Several studies, mostly in Western countries, show that in an observational, real-life setting, higher consumption of dairy products is associated with improved diet quality and higher intakes of key nutrients like calcium and vitamin D [12,13,14]. An Australian study [12] found that children aged 8 to 10 years with higher dairy consumption had significantly higher intakes of energy, protein, calcium, phosphorus, magnesium, potassium, zinc, and vitamins A, B_2_, and B_3_, while their intakes of mono- and polyunsaturated fats were lower. In addition, higher dairy consumption was associated with higher consumption of cereals and grains and reduced intake of energy-dense, nutrient-poor foods. The Australian study further showed that children who consumed two or more servings of dairy per day (38%) were more likely to meet food and nutrient recommendations, and milk intake was inversely associated with the intake of sugar-sweetened beverages. Children who did not meet their minimum dairy serving recommendations consumed higher quantities of sugar-sweetened beverages than milk. Similarly, a study in children aged 7 to 13 years in the Netherlands reported that higher dairy consumption was associated with significantly higher intakes of fruits, vegetables, and cereals and lower consumption of soft drinks and fruit juices. Also, in this study, children in the higher dairy consumption tertiles had significantly higher intakes of, e.g., energy, protein, calcium, iodine, phosphorus, magnesium, potassium, zinc, retinol equivalents, and vitamin B_2_, B_12_, and D [13]. Furthermore, the SEANUTS I study among children aged 0.5–12 years in Indonesia, Malaysia, Thailand, and Vietnam found a positive association of dairy consumption with energy, protein, calcium, iron, and zinc, as well as with vitamin B_1_, B_2_, B_3_, C, A, and D [14].

To evaluate public health relevance of a staple food product like dairy, it is important to conduct analyses within the local context using recent, population-representative dietary surveys. For Asia, there is one such analysis available on dairy in the SEANUTS I of 2010–2011 [14]. In this study, associations were expressed per dairy serving consumed but lacked information on the impact of meeting dairy recommendations. Furthermore, no information was provided on the association of dairy intake with foods from other product categories. In addition, for Malaysia, nutrient intake data were derived from food frequency instead of 24 h recall, which is often preferred for assessing nutrient intake.

Our study aimed to assess the associations of different dairy consumptions, either categorized in quartiles or based on dairy recommendations, with nutrient intake (adequacy) among children aged 2 to 12 years in Indonesia, Malaysia, Thailand, and Vietnam. Our study aims to examine the impact of dairy consumption on food, energy, and nutrient intake among children aged 2 to 12 years in Indonesia, Malaysia, Thailand, and Vietnam, using recent population-representative dietary surveys.

## 2. Materials and Methods

### 2.1. Study Design, Sample Size and Participants

This sub-analysis is part of the South East Asian Nutrition Surveys (SEANUTS) II (*n* = 13,933), a multi-center, cross-sectional survey conducted from May 2019 to April 2021 in Indonesia (*n* = 3465), Malaysia (*n* = 2989), Thailand (*n* = 3478), and Vietnam (*n* = 4001) that aimed to gather comprehensive nutritional data from children aged 6 months to 12 years [15]. The sample size calculation for the original SEANUTS II is explained in the paper, outlining its rationale and study design [15]. For each country, total sample size was determined based on national prevalence data for stunting, overweight, anemia, and deficiencies in vitamin A, vitamin D, or zinc, depending on the most relevant nutritional concerns.

### 2.2. Inclusion and Exclusion Criteria

The inclusion criteria of this sub-study specified that only apparently healthy children aged 2 to 12 years, who were citizens of the studied countries and for whom valid data had been collected, were included. Exclusion criteria were signs of physical disability; genetic, cardiovascular, or respiratory illness that limited physical activity; medical history or illness within the past 3 months; feeling unwell on the day of measurement; and implausible energy intake determined from the 24 h dietary recall. Recruitment was limited to one child per household.

### 2.3. Dietary Intake Assessment and Definition of Dairy

For the primary objective, we used a one-day 24 h dietary recall. The 24 h recall was collected via (1) parent-proxy face-to-face interviews with mothers or main caregivers for children aged 0.5 to 10 years and (2) combined child self-report and parent-proxy face-to-face interviews for children aged 10 to 12 years.

To identify and exclude implausible energy intake data from the 24 h dietary recall in Indonesia, Malaysia, and Vietnam, we computed the ratio of reported energy intake to predicted energy expenditure based on the Black and Cole formula [16]. Energy expenditure was estimated by multiplying basal metabolic rate [17] by a moderate physical activity level [18]. Subsequently, we applied a 99% confidence interval in the Black and Cole formula, accounting for an 8.2% within-subject variation in energy expenditure and a 23% within-subject variation in energy intake [19]. Based on this 99% confidence interval, participants with an energy intake to energy expenditure ratio falling within 0.27–1.73 were included in the analysis. In Thailand, implausible energy intake data were assessed using the predicted total energy expenditure (pTEE) method [20]. Plausible reporters were identified based on the ratio of reported intakes to pTEE. Energy expenditure was predicted based on a moderate physical activity level [21]. The standard deviation (SD) was derived from the coefficient of variation (CV) of the reported energy intake within the study population and the CV of pTEE [21]. The confidence interval limits for plausible reported energy intakes were defined as the pTEE ± 2 SD. As the SEANUTS II study did not include a structural assessment of supplement use (e.g., via a supplement-specific questionnaire), supplement intake could not be incorporated into nutrient intake calculations.

Food items were categorized into five food groups according to local dietary guidelines (cereals and grains, vegetables, fruits, dairy, and meat and protein foods) and extra foods, which encompassed food items not categorized within the other food groups [11]

Dairy encompassed liquid and powdered animal-based dairy products, excluding human breast milk and plant-based alternatives. Condensed milk, butter, cream, ice cream, and sour milk were not considered dairy. Stage 1 and 2 formulas, were classified as Infant Formula and Toddler (IFT)stage 1 and 2, and formula of 3 and higher were classified as Young Child Formula (YCF). Standardized serving sizes for dairy products were established according to regional recommendations. Table 1 presents the dairy recommendations and serving sizes used per country.

Further details on serving sizes for other food items and daily food group recommendations can be found in the following reports [22,23,24,25]. Dairy intake was categorized into tertiles (low consumers with <1 serving in all countries except Vietnam with <2 servings; middle consumers with 1–2.5 servings in Indonesia, 1–2 servings in Malaysia and Thailand, and 2–3 servings in Vietnam; and high consumers with >2.5 servings in Indonesia, >2 servings in Malaysia and Thailand, and >3 servings in Vietnam) and non-consumers (0 servings).

### 2.4. Statistical Analyses

Each country’s data was analyzed separately. Statistical analyses were conducted using the R statistical programming environment (version 4.4.2). We used the pastecs package and tbl_summary package (version 4.4.2) for descriptive statistics. To check assumptions, data normality in each country and subgroup was assessed using the Shapiro–Wilk W test. Homogeneity of variance was checked using Levene’s test from the car package. Results are presented as medians (interquartile range [IQR]). Chi-square tests were performed using the gmodels package (version 4.4.2) to compare frequencies in categorical data if expected frequencies were above 5; otherwise, Fisher’s exact tests were conducted. In cases where the overall test for more than two groups was significant, standardized residuals were calculated to identify which groups contributed to the significant result. To make comparisons between two groups with continuous variables as outcomes, Wilcoxon’s rank-sum tests were used. For comparisons between more than two groups, Kruskal–Wallis tests with post hoc tests (which were corrected for the number of tests and for which the pgirmess package (version 4.4.2) was used) were used. We analyzed each country’s data separately. Food and nutrient intake data were stratified by age group (2 to 3 years, 4 to 6 years, and 7 to 12 years).

## 3. Results

### 3.1. Participants

**Figure 1:**
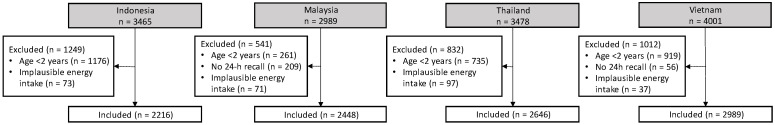
Participant flow chart for children included to assess the impact of dairy consumption on energy and nutrient intake.

A total of 1249 children in Indonesia, 541 in Malaysia, 832 in Thailand, and 1012 in Vietnam were excluded from the analysis. Of these, 73, 71, 97, and 37 children, respectively, in each country, were excluded due to implausible energy intake values. The final analytic sample comprised 2216 children in Indonesia, 2448 in Malaysia, 2646 in Thailand, and 2989 in Vietnam (Figure 1). The mean age of the children included was 6.7 years in Indonesia, 7.6 years in Malaysia, 6.6 years in Thailand, and 6.8 years in Vietnam. Girls accounted for 52% of the population in Indonesia and Malaysia, 50% in Thailand, and 49% in Vietnam. The proportion of children living in rural areas was 47% in Indonesia, 30% in Malaysia, 68% in Thailand, and 70% in Vietnam. Poverty prevalence among children was 35% in Indonesia, 15% in Malaysia, 29% in Thailand, and 5% in Vietnam.

### 3.2. Consumption of Dairy

In Indonesia, Malaysia, Thailand, and Vietnam, 39.0%, 56.3%, 73.4%, and 56.5% of children, respectively, consumed dairy, of which 23.8%, 17.3%, 23.4%, and 8.0% met the daily dairy recommendation. The recommended number of daily dairy servings is one serving in Indonesia [22], two in Malaysia [23] and Thailand [24], and four servings for children aged 1 to 9 years and six servings for children aged 10 to 12 years in Vietnam [25]. In the overall sample, among dairy consumers (which excludes the non-consumers), the median (IQR) daily dairy intake was 1.4 (0.6–3.0) servings in Indonesia, 1.2 (0.8–2.4) servings in Malaysia, 1.8 (1.0–2.7) servings in Thailand, and 2.1 (1.6–3.5) servings in Vietnam. One serving of dairy corresponded to 200 mL of milk in Indonesia and Thailand, 250 mL in Malaysia, and 100 mL in Vietnam. In Indonesia, the highest consumption of dairy products was for milk at 55.3%, followed by YCF at 31.7%, and IFT stage 1 and 2 at 12.7%. Cheese consumption was minimal at 0.3%, while yogurt consumption was nonexistent. In Malaysia, the highest consumption of dairy products was for YCF at 62.8%, followed by milk at 33.9%, and cheese at 2.7%. IFT stage 1 and 2 and yogurt consumption was minimal at 0.8% and 0.7%, respectively. In Thailand and Vietnam, the highest consumption of dairy products was for milk at 89.0% and 78.5%, respectively, followed by YCF at 9.7% and 20.2%, respectively, and yogurt at 1.0% and 8.6%, respectively. IFT stage 1 and 2 (0.8% and 0.9%, respectively) and cheese consumption was minimal (both 0.4%).

Table 2 presents the sociodemographic characteristics of children consuming high-, middle-, low-, and no amount of dairy as well as children meeting and not meeting the daily dairy recommendations across all four countries. Fewer children consumed dairy in the older group, with the highest proportion of children consuming dairy observed in the youngest and the lowest in the oldest age group, and more children from the younger age groups were high- and middle-dairy consumers compared to low and no consumers (*p* < 0.001). Consistently, a higher proportion of younger children met the daily dairy recommendations compared to older children (*p* < 0.001). There were no significant differences in dairy consumption and meeting versus not meeting the daily dairy recommendations between female and male, except in Vietnam, where more female middle-dairy consumers were observed compared to males (*p* < 0.01), and where more male children were likely to meet the recommendations (*p* < 0.01). In Indonesia, Malaysia, and Vietnam, there were higher proportions of children living in urban areas and middle-dairy consumers compared to children living in rural areas (*p* < 0.001), while in Thailand, there was no significant difference in dairy consumption between residential areas. Consistently, higher proportions of children living in urban areas in Indonesia, Malaysia, and Vietnam met the daily dairy recommendations (*p* < 0.001, except Malaysia *p* < 0.05), with no differences in Thailand. Higher income groups consistently showed higher dairy consumption and a higher proportion of children meeting the daily dairy recommendations compared to lower income groups in all countries (*p* < 0.001, except Thailand *p* < 0.01).

### 3.3. Food Intakes by Dairy Consumption Categories and by Meeting Daily Dairy Recommendations

Table 3 presents food intakes of food groups other than dairy among high-, middle-, low-, and non-dairy consuming children. Overall intake of fruits and vegetables was very low, mostly below 0.5 servings a day for all levels of dairy consumers. For all four countries, when comparing high-dairy consumers with non-dairy consumers, the former consumed approximately 1 serving less of cereals and grains and 0.5 serving less of meat and protein foods (*p* < 0.01 for all comparisons). Furthermore, compared to non-dairy consumers, high-dairy consumers ate significantly less extra foods: 1.7, 2.5, 1.8, and 0.6 less servings, for Indonesia, Malaysia, Thailand, and Vietnam, respectively (*p* < 0.001 for all comparisons).

The same patterns were observed when comparing children who met the dairy recommendations to children not meeting the dairy recommendations. Those meeting and not meeting dairy recommendations had statistically significant different intake of cereals and grains, meat and protein foods, and extra foods, for all four countries.

Also, in the age-stratified analyses (supplementary Table S1), the association with extra foods stood out. In all countries, a high dairy intake was significantly associated with a lower intake of extra foods in children aged 2–3 years old. This was also the case for 4–6-year-old children in Malaysia and Thailand and for 7–12-year-old children in Thailand and Vietnam.

### 3.4. Energy and Nutrient Intakes by Dairy Consumption Categories and by Meeting Daily Dairy Recommendations

Table 4 presents the daily energy and nutrient intakes among high-, middle-, low-, and non-dairy consuming children in the overall sample of Indonesia, Malaysia, Thailand, and Vietnam.

Overall, for all four countries, when comparing high-dairy consumers with non-dairy consumers, the former had higher intakes of energy and macro- and micronutrients. There were a few exceptions: In Thailand, energy intake was only higher in age stratified analysis, and zinc, iron, and magnesium were only higher in the subgroup of 2–3-year-old children (supplementary Table S2). Furthermore, no clear differences were found with vitamin B_3_, sodium, and vitamin C in any age category. Carbohydrate intake was higher among non-dairy consumers in the subgroup of children aged 2–3 years but not in the other subgroups. For Malaysia, non-dairy consumers only had higher sodium intake among the 7–12-year-olds. In addition, in Vietnam, high-dairy consumers only had a higher carbohydrate intake in the age stratified analyses.

When comparing high-dairy consumers with low-dairy consumers in Indonesia, intakes of energy and all nutrients were higher, except for fiber and choline, which did not differ between the groups. In Malaysia, intake of micronutrients was higher in high-dairy consumers compared to low-dairy consumers, except for intake of β-carotene which did not differ between the two groups. Similarly, no differences were found for energy and the macronutrients. In contrast, sodium intake was higher in low-dairy consumers compared to high-dairy consumers in 7–12-year-olds. In Vietnam, energy and nutrient intakes were higher in the high-dairy group compared with the low-dairy group, except for intakes of carbohydrate, vitamin C, and iron, which did not differ. In Thailand, the patterns were more mixed and similar to the comparison with non-dairy consumers. Additionally, in the comparison of high- with low-dairy consumers, no differences were found with intakes of protein, fiber, and vitamin B_1_.

Similar patterns were observed when comparing children who met the daily dairy recommendations to children not meeting these recommendations (Table 4; Supplementary Table S2).

Next to higher nutrient intakes, high-dairy consumers were also more likely to meet the EER and RNI for energy and macro- and micronutrients. For example, 69.6% of the Indonesian high-dairy consumers met the RNI for calcium compared to only 0.9% in the group of non-dairy consumers. For Malaysia, Thailand, and Vietnam, the values were 64.3% vs. 1.7%, 77.4% vs. 3.3%, and 59.4% vs. 2.2%, respectively. While most children in the high-dairy consumer group were meeting RNI for most nutrients, this was not the case for potassium and vitamin D. For potassium, 0.2% of the Malaysian high-dairy consumers and 19.7% of the Thai high-dairy consumers met the RNI. For vitamin D, only 22.7%, 20.1%, 4.0%, and 17.3% of the high-dairy consumers in Indonesia, Malaysia, Thailand, and Vietnam, respectively, were meeting the RNI. At the same time, hardly any of the non-dairy consumers met the RNI for vitamin D. The same patterns were observed when comparing the group meeting daily dairy recommendations with the group not meeting these recommendations. The percentage meeting calcium recommendations among those meeting the dairy recommendations were 49.4%, 63.8%, 77.3%, and 90.0% for Indonesia, Malaysia, Thailand, and Vietnam, respectively. Among those not meeting dairy recommendations, the percentage meeting calcium RNI was low for all four countries and ranged from 4.5% for Indonesia to 9.1% for Thailand.

## 4. Discussion

These analyses of the SEANUTS II study provide a comprehensive overview of dairy consumption and its association with food and nutrient intakes among children aged 2.0–12.9 years in Indonesia, Malaysia, Thailand, and Vietnam. Across these countries, dairy consumption was low, particularly among children aged 4 years and older. In general, children with a higher dairy intake consumed less food from non-dairy food groups, mainly extra (non-basic) foods. Consistently, higher dairy consumption was associated with increased energy and nutrient intakes, including protein, vitamins A, B_2_, B_12_, D, and calcium.

Consistent with previous research [14], dairy intake was low in Indonesia, Malaysia, Thailand, and Vietnam. The percentages of children (aged 2–12) meeting the local dairy recommendations ranged from 8% in Vietnam to 24% in Indonesia. The percentages were lower in younger (2–3 years) children and higher in older (7–12 years) children. Among children aged 7–12 years, less than 10% met the dairy recommendations in all four countries. Several factors may contribute to the low dairy consumption, including the absence of dairy in traditional diets, limited awareness of dairy’s benefits, taste, and affordability [26], and competition from other beverages such as malted drinks [27]. Higher dairy consumption was more prevalent among high-income children, highlighting the role of affordability. In Indonesia, the relatively low national recommendation (200 mL/day) compared with ≥400 mL/day in other countries may partly explain the higher proportion of children meeting local guidelines.

The prevalence of children who did not consume any dairy during the 24 h recall was high, ranging from 26.6% in Thailand to 61.0% in Indonesia. Thailand’s lower proportion may reflect the extensive school milk program, which has been in place since 1992 [28]. Non-consumption was most pronounced among older children, which may be linked to the high prevalence of lactase non-persistence (LNP) in Asia. Although LNP reduces lactase production after weaning, evidence suggests that moderate milk intake (one to two glasses daily) is generally tolerated when introduced gradually, likely explained by increased levels of Bifidobacterium, which can metabolize lactose without gas production [29]. Misconceptions about lactose intolerance may, therefore, contribute to avoidance of dairy in older children and explain the high prevalence of non-consumers of dairy among older children.

Beyond dairy, children in the SEANUTS II study also exhibited insufficient consumption of basic food groups. Consistent with findings from previous research [30,31], intakes of fruits and vegetables were notably low, often averaging to less than half a serving a day. These low intakes were observed both in children meeting and not meeting the dairy recommendations. The most pronounced dietary differences across dairy intake groups (categorized as none, low, middle, and high) were found in the intake of “extra foods” (such as snacks and sugar-sweetened beverages). In line with the inverse association of dairy with extra foods observed in SEANUTS II, studies conducted in Australia among children aged 8–10 years [12] and in the Netherlands among children aged 7–13 years [13] identified an inverse association of milk intake with, respectively, extra foods and sugar-sweetened beverages. These findings are also consistent with the observation of Nicklas et al. [26] who identified that one of the core barriers for dairy intake in children is competing foods, such as sugar-sweetened beverages.

In all four countries, higher dairy consumption was associated with increased energy and nutrient intakes, including protein, vitamins A, B_2_, B_12_, D, and calcium. These findings are consistent with prior observational and clinical studies, which show that dairy consumption positively impacts micronutrient intake and status [12,13,32,33,34,35]. Furthermore, results align with a modeling study [33] in which substitution of all none-milk caloric beverages with milk in the diets of USA children resulted in increased intakes of, among others, protein, calcium, potassium, sodium, and vitamins A, B_12_, and D.

In line with the aforementioned findings, the vast majority of high-dairy consumers had adequate intakes for most nutrients, including protein, vitamins A, B_2_, B_12_, D, and calcium. In contrast, the prevalence of inadequate nutrient intakes was much higher among children with no or low dairy consumption. For example, for calcium, fewer than 10% of low- or non-dairy consumers met the RNI in all four countries, compared with 59.4–77.4% of high-dairy consumers. These results substantiate the role of dairy in securing adequate calcium provision, a key determinant of bone mineral content [36]. Moreover, as several intervention studies have shown the effect of milk on bone mineral content and linear growth [1,4], this observation underscores the importance of adequate dairy intake for bone development in SEA children. Similarly, for Indonesia, 93% of high-dairy consumers met the RNI for protein, whereas 43.7% of non-dairy consumers met the RNI. This observation underlines the role dairy has in providing sufficient, high-quality protein. High-quality (milk) proteins are well-known for their growth-promoting effect [37]. This is especially relevant in the Indonesian context where over 20% of children aged 12 years and below are stunted [10].

In accordance with the results of the four respective dairy consumer groups, children meeting dairy recommendations generally had higher nutrient intakes and lower prevalences of nutrient inadequacies, though absolute levels varied by country. These differences between countries largely reflect differences in average nutrient intakes, which might be explained by cultural and programmatic factors unique to each country. However, in the case of calcium, disparities appeared to be attributable to differences in national dietary guidelines. The dairy recommendation for Indonesia of 1 serving of dairy per day [22] was notably lower than the recommendations in Malaysia [23] and Thailand [24], which recommended 2 servings a day, or Vietnam, which recommended 4–6 small (100 mL) servings [25]. Accordingly, among children meeting the dairy recommendation, the prevalence of children not meeting the RNI for calcium was lowest in Indonesia (49.4%), followed by Malaysia (63.8%), Thailand (77.3%), and Vietnam (90.0%). Nonetheless, even among children meeting dairy recommendations, fiber and potassium intakes remained insufficient, highlighting the need to consume a balanced diet with a variety of nutrient-rich food groups such as fruits, vegetables, and whole-grains alongside dairy. These observations also suggest opportunities for dietary interventions, such as the development of a breakfast option that combines dairy with (cereal) fiber and possibly (fresh) fruits and vegetables.

A strength of this study is its large sample size encompassing over 10,000 children across four different Southeast Asian countries. Furthermore, the use of a population-representative sampling strategy ensures applicability of the results to the general population. However, for Malaysia, only children from Peninsular Malaysia and for Indonesia, only children from mainly Java and Sumatera islands were included, due to COVID’s interference with data collection. Notably, this study focused on the Southeast Asian region, whereas most other studies focused on Western countries. Additionally, the inclusion of both food and nutrient intakes provided a comprehensive picture of children’s dietary habits. However, certain limitations should be acknowledged. First, the cross-sectional nature of this study precludes any inference on causality. Second, use of a single instead of multiple 24 h dietary recalls, precludes the correction for intra-individual variability of dietary intake data. The latter may lead to an overestimation of the number of non-consumers and random misclassification of dairy intake categories. However, it is unlikely to bias comparisons, e.g., significantly impact the proportions of children meeting the dairy recommendations. Third, under-reporting is an inherent limitation of the 24 h dietary recall method, and this risk is further increased when intake data are proxy-reported by parents or caregivers, as some eating occasions may occur without their presence (e.g., in childcare centers or schools). Finally, the absence of systematically collected dietary supplement data may have influenced estimates of overall nutrient intake among children.

## 5. Conclusions

Dairy consumption among Southeast Asian children was generally low, particularly in older age groups. Less than 10% of children aged 7–12 years in Indonesia, Malaysia, Thailand, and Vietnam met the dairy intake recommendations. Higher dairy intake was associated with lower intake of extra foods and a higher proportion of children meeting recommended nutrient intakes of, e.g., zinc, calcium, and vitamins A, B_2_, B_12_, and D. Among children meeting the dairy recommendations, the majority had adequate intakes for key dairy nutrients like vitamin B_12_ and protein. Furthermore, most children meeting the dairy recommendations met the RNI for calcium in Malaysia, Thailand, and Vietnam. These findings underscore the important role of dairy in nutrient provision among Southeast Asian children. Furthermore, the generally low intakes of potassium and fiber in all children, independent of dairy consumption level, emphasize the need to increase the intake of other basic product groups, such as fruits and vegetables. To safeguard nutrient adequacy in Southeast Asian children, it is, therefore, recommended to implement public health policies which sustain dairy consumption in older children at approximately two servings a day as well as increase the consumption of fruit and vegetables. Continued monitoring of dietary intake in Southeast Asian children is warranted, ideally using at least two 24 h recalls to improve accuracy. Furthermore, even though the focus of most nutritional research is on nutrient intake from food sources, additional systematic assessment on supplement use would provide a more comprehensive understanding of total nutrient intake.

## Figures and Tables

**Table 1:** Overview of regional dairy recommendations and serving sizes.

Country	Dairy Recommendation	Serving Size
Indonesia [22]	1 serving	200 mL milk 20 g milk powder 185 mL goat milk 40 g cheese
Malaysia [23]	2 servings	250 mL milk 30 g milk powder 40 g cheese 135 g Greek yogurt
Thailand [24]	2 servings	200 mL milk 150 g yogurt 30 g cheese
Vietnam [25]	4–6 servings	100 mL milk 15 g milk powder 100 g yogurt 15 g cheese

**Table 2:** Sociodemographic characteristics of children by dairy intake category and by meeting and not meeting daily dairy recommendations in Indonesia, Malaysia, Thailand, and Vietnam.

	High-Dairy Consumer	Middle-Dairy Consumer	Low-Dairy Consumer	Non-Dairy Consumer	*p*-Value	Meeting Daily Dairy Recommendation	Not Meeting Daily Dairy Recommendation	*p*-Value
**Indonesia**, *n* (%)	286 (12.9%)	242 (10.9%)	337 (15.2%)	1351 (61.0%)		528 (23.8%)	1688 (76.2%)	
Age, *n* (%)								
2–3 years	170 (27.0%) ^+^	111 (17.6%) ^+^	116 (18.4%) ^+^	232 (36.9%) ^−^	<0.001	281 (44.7%) ^+^	348 (55.3%) ^−^	<0.001
4–6 years	91 (17.4%) ^+^	54 (10.3%)	96 (18.4%)	281 (53.8%) ^−^		145 (27.8%)	377 (72.2%)	
7–12 years	25 (2.3%) ^−^	77 (7.2%) ^−^	125 (11.7%) ^−^	838 (78.7%) ^+^		102 (9.6%) ^−^	963 (90.4%) ^+^	
Sex, *n* (%)								
Female	141 (12.3%)	120 (10.5%)	169 (14.8%)	715 (62.4%)	0.53	261 (22.8%)	884 (77.2%)	0.24
Male	145 (13.6%)	122 (11.4%)	168 (15.7%)	636 (59.4%)		267 (24.9%)	804 (75.1%)	
Area, *n* (%)								
Urban	183 (15.7%) ^+^	155 (13.3%) ^+^	157 (13.4%)	673 (57.6%)	<0.001	338 (28.9%)	830 (71.1%)	<0.001
Rural	103 (9.8%) ^−^	87 (8.3%) ^−^	180 (17.2%)	678 (64.7%)		190 (18.1%)	858 (81.9%)	
Income group, *n* (%)								
High-income	10 (23.8%) ^+^	7 (16.7%)	4 (9.5%)	21 (50.0%)	<0.001	17 (40.5%) ^+^	25 (59.5%)	<0.001
Middle-income	81 (20.9%) ^+^	62 (16.0%) ^+^	58 (15.0%)	186 (48.1%) ^−^		143 (37.0%) ^+^	244 (63.0%) ^−^	
Low-income	144 (14.4%)	117 (11.7%)	162 (16.2%)	578 (57.7%)		261 (26.1%)	740 (73.9%)	
Poor	51 (6.5%) ^−^	56 (7.1%) ^−^	113 (14.4%)	566 (72.0%) ^+^		107 (13.6%) ^−^	679 (86.4%) ^+^	
**Malaysia**, *n* (%)	417 (17.0%)	423 (17.3%)	538 (22.0%)	1070 (43.7%)		423 (17.3%)	2025 (82.7%)	
Age, *n* (%)								
2–3 years	179 (54.7%) ^+^	75 (22.9%) ^+^	32 (9.8%) ^−^	41 (12.5%) ^−^	<0.001	180 (55.0%) ^+^	147 (45.0%) ^−^	<0.001
4–6 years	189 (24.6%) ^+^	155 (20.2%)	170 (22.1%)	255 (33.2%) ^−^		190 (24.7%) ^+^	579 (75.3%) ^−^	
7–12 years	49 (3.6%) ^−^	193 (14.3%) ^−^	336 (24.9%) ^+^	774 (57.2%) ^+^		53 (3.9%) ^−^	1299 (96.1%) ^+^	
Sex, *n* (%)								
Female	201 (15.8%)	206 (16.2%)	280 (22.0%)	583 (45.9%)	0.08	204 (16.1%)	1066 (83.9%)	0.10
Male	216 (18.3%)	217 (18.4%)	258 (21.9%)	487 (41.3%)		219 (18.6%)	959 (81.4%)	
Area, *n* (%)								
Urban	310 (18.2%)	330 (19.4%) ^+^	375 (22.0%)	689 (40.4%) ^−^	<0.001	315 (18.5%)	1389 (81.5%)	<0.05
Rural	107 (14.4%)	93 (12.5%) ^−^	163 (21.9%)	381 (51.2%) ^+^		108 (14.5%)	636 (85.5%)	
Income group, *n* (%)								
High-income	174 (20.4%) ^+^	181 (21.3%) ^+^	171 (20.1%)	325 (38.2%) ^−^	<0.001	176 (20.7%) ^+^	675 (79.3%)	<0.001
Middle-income	70 (15.9%)	72 (16.4%)	111 (25.3%)	186 (42.4%)		71 (16.2%)	368 (83.8%)	
Low-income	124 (16.4%)	115 (15.3%)	171 (22.7%)	344 (45.6%)		127 (16.8%)	627 (83.2%)	
Poor	37 (10.3%) ^−^	52 (14.5%)	78 (21.7%)	192 (53.5%) ^+^		37 (10.3%) ^−^	322 (89.7%)	
Missing values	12 (26.7%)	3 (0.1%)	7 (15.6%)	23 (51.1%)		12 (26.7%)	33 (73.3%)	
**Thailand**, *n* (%)	619 (23.4%)	527 (19.9%)	796 (30.1%)	704 (26.6%)		620 (23.4%)	2026 (76.6%)	
Age, *n* (%)								
2–3 years	376 (51.9%) ^+^	170 (23.5%) ^+^	96 (13.3%) ^−^	82 (11.3%) ^−^	<0.001	376 (51.9%) ^+^	348 (48.1%) ^−^	<0.001
4–6 years	173 (20.5%)	214 (25.3%) ^+^	296 (35.1%) ^+^	161 (19.1%) ^−^		174 (20.6%)	671 (79.4%)	
7–12 years	70 (6.5%) ^−^	143 (13.3%) ^−^	404 (37.4%) ^+^	461 (42.8%) ^+^		70 (6.5%) ^−^	1007 (93.5%) ^+^	
Sex, *n* (%)								
Female	321 (24.5%)	263 (20.1%)	391 (29.8%)	336 (25.6%)	0.50	322 (24.6%)	989 (75.4%)	0.17
Male	298 (22.3%)	264 (19.8%)	405 (30.3%)	368 (27.6%)		298 (22.3%)	1037 (77.7%)	
Area, *n* (%)								
Urban	192 (23.2%)	167 (20.2%)	248 (30.0%)	221 (26.7%)	1.00	193 (23.3%)	635 (76.7%)	0.92
Rural	427 (23.5%)	360 (19.8%)	548 (30.1%)	483 (26.6%)		427 (23.5%)	1391 (76.5%)	
Income group, *n* (%)								
High-income	76 (29.7%) ^+^	57 (22.3%)	72 (28.1%)	51 (19.9%) ^−^	<0.01	76 (29.7%) ^+^	180 (70.3%)	<0.01
Middle-income	85 (28.3%)	62 (20.7%)	73 (24.3%)	80 (26.7%)		85 (28.3%)	215 (71.7%)	
Low-income	306 (23.1%)	263 (19.9%)	387 (29.3%)	366 (27.7%)		307 (23.2%)	1015 (76.8%)	
Poor	151 (19.7%) ^−^	145 (18.9%)	264 (34.4%) ^+^	207 (27.0%)		151 (19.7%) ^−^	616 (80.3%)	
Missing values	1 (100%)	0 (0%)	0 (0%)	0 (0%)		1 (100%)	0 (0%)	
**Vietnam**, *n* (%)	556 (18.6%)	357 (11.9%)	777 (26.0%)	1299 (43.5%)		239 (8.0%)	2750 (92.0%)	
Age, *n* (%)								
2–3 years	273 (39.7%) ^+^	151 (21.9%) ^+^	155 (22.5%)	109 (15.8%) ^−^	<0.001	137 (19.9%) ^+^	551 (80.1%) ^−^	<0.001
4–6 years	150 (19.7%)	107 (14.1%)	227 (29.9%) ^+^	276 (36.1%) ^−^		60 (7.9%)	700 (92.1%)	
7–12 years	133 (8.6%) ^−^	99 (6.4%) ^−^	395 (25.6%)	914 (59.3%) ^+^		42 (2.7%) ^−^	1499 (97.3%) ^+^	
Sex, *n* (%)								
Female	250 (16.9%)	205 (13.9%) ^+^	376 (25.4%)	648 (43.8%)	<0.01	99 (6.7%)	1380 (93.3%)	<0.01
Male	306 (20.3%)	152 (10.1%) ^−^	401 (26.6%)	651 (43.1%)		140 (9.3%)	1370 (90.7%)	
Area, *n* (%)								
Urban	189 (21.2%)	134 (15.0%) ^+^	250 (28.0%)	320 (35.8%) ^−^	<0.001	95 (10.6%)	798 (89.4%)	<0.001
Rural	367 (17.5%)	223 (10.6%)	527 (25.1%)	979 (46.7%) ^+^		144 (6.9%)	1952 (93.1%)	
Income group, *n* (%)								
High-income	105 (20.5%)	75 (14.6%)	159 (31.0%) ^+^	174 (33.9%) ^−^	<0.001	51 (9.9%)	462 (90.1%)	<0.001
Middle-income	358 (21.3%) ^+^	212 (12.6%)	451 (26.8%)	659 (39.2%) ^−^		152 (9.0%)	1528 (91.0%)	
Low-income	89 (13.9%) ^−^	67 (10.5%)	151 (23.6%)	332 (52.0%) ^+^		34 (5.3%) ^−^	605 (94.7%)	
Poor	4 (2.5%) ^−^	3 (1.9%) ^−^	16 (10.2%) ^−^	134 (85.4%) ^+^		2 (1.3%) ^−^	155 (98.7%)	

Data show frequencies and proportions (%). Chi-square tests were conducted within each stratification group. ^+^ significantly more than expected based on standardized residuals > 1.96. ^−^−significantly less than expected based on standardized residuals < −1.96.

**Table 3:** Food intake, among high-, middle-, low-, and non-dairy consumers as well as among children meeting and not meeting the daily dairy recommendations.

Food Intake (Servings/Day)	High-Dairy Consumer	Middle-Dairy Consumer	Low-Dairy Consumer	Non-Dairy Consumer	*p*-Value	Meeting Daily Dairy Recommendation	Not Meeting Daily Dairy Recommendation	*p*-Value
**Indonesia**, *n*	286	242	337	1351		528	1688	
Cereals and grains	2.0 (1.3, 3.0) ^a^	2.5 (1.5, 4.0) ^b^	2.5 (1.5, 4.0) ^b^	3.0 (2.0, 4.7) ^c^	<0.001	2.2 (1.5, 3.5)	2.9 (1.8, 4.5)	<0.001
Fruits	0.0 (0.0, 0.8)	0.0 (0.0, 0.9)	0.0 (0.0, 1.2)	0.0 (0.0, 1.0)	0.02	0.0 (0.0, 0.8)	0.0 (0.0, 1.0)	0.46
Vegetables	0.2 (0.0, 0.5)	0.2 (0.0, 0.5)	0.2 (0.0, 0.5)	0.2 (0.0, 0.6)	0.12	0.2 (0.0, 0.5)	0.2 (0.0, 0.6)	0.17
Meat and proteinaceous foods	2.1 (1.3, 3.2) ^a^	2.5 (1.6, 3.6) ^b^	2.6 (1.6, 3.6) ^b^	2.5 (1.6, 3.7) ^b^	0.002	2.3 (1.5, 3.4)	2.5 (1.6, 3.7)	0.008
Extra foods	5.6 (3.3, 8.4) ^a^	6.6 (4.4, 9.9) ^b^	7.1 (4.4, 10.2) ^b^	7.3 (4.9, 10.5) ^b^	<0.001	6.2 (3.6, 9.0)	7.2 (4.9, 10.4)	<0.001
**Malaysia**, *n*	417	423	538	1070		423	2025	
Cereals and grains	2.7 (1.8, 3.6) ^a^	3.4 (2.4, 4.6) ^b^	3.5 (2.6, 4.8) ^b^	3.5 (2.5 4.8) ^b^	<0.001	2.7 (1.8, 3.6)	3.5 (2.5, 4.7)	<0.001
Fruits	0.1 (0.0, 1.0) ^a^	0.3 (0.0,1.0) ^a,b^	0.2 (0.0, 0.9) ^a^	0.0 (0.0 0.8) ^a,c^	0.001	0.1 (0.0, 1.0)	0.0 (0.0, 0.9)	0.72
Vegetables	0.4 (0.0, 1.3) ^a^	0.7 (0.0, 1.8) ^b^	0.7 (0.0, 1.9) ^b,c^	0.4 (0.0 1.4) ^a^	<0.001	0.4 (0.0, 1.3)	0.6 (0.0, 1.6)	0.14
Meat and proteinaceous foods	1.0 (0.5, 1.6) ^a^	1.4 (0.9, 2.2) ^b^	1.4 (0.8, 2.2) ^b^	1.5 (0.9 2.3) ^b^	<0.001	1.0 (0.5, 1.6)	1.4 (0.9, 2.3)	<0.001
Extra foods	2.5 (1.4, 4.2) ^a^	3.2 (2.0, 6.1) ^b^	4.3 (2.4, 7.3) ^c^	5.0 (2.8 8.2) ^d^	<0.001	2.5 (1.4, 4.3)	4.5 (2.5, 7.6)	<0.001
**Thailand**, *n*	619	527	796	704		620	2026	
Cereals and grains	2.2 (1.4, 3.1) ^a^	2.7 (1.9, 4.1) ^b^	3.1 (2.2, 4.3) ^c^	3.3 (2.2, 4.5) ^c^	<0.001	2.2 (1.4, 3.2)	3.1 (2.1, 4.3)	<0.001
Fruits	0.3 (0.0, 1.3) ^a^	0.3 (0.0, 1.3) ^a^	0.3 (0.0, 1.3) ^a^	0.0 (0.0, 0.9) ^b^	<0.001	0.3 (0.0, 1.3)	0.2 (0.0, 1.1)	0.54
Vegetables	0.4 (0.0, 0.9) ^a^	0.5 (0.0, 1.2) ^b^	0.7 (0.1, 1.5) ^c^	0.4 (0.0, 1.2) ^a,b^	<0.001	0.4 (0.0, 0.9)	0.5 (0.0, 1.3)	<0.001
Meat and proteinaceous foods	3.7 (2.2, 5.6) ^a^	4.3 (2.8, 5.9) ^b^	4.2 (2.9, 6.1) ^b^	4.4 (2.8, 6.4) ^b^	<0.001	3.7 (2.2, 5.5)	4.3 (2.8, 6.1)	<0.001
Extra foods	1.5 (0.7, 3.0) ^a^	2.0 (1.0, 3.5) ^b^	2.9 (1.6, 4.6) ^c^	3.3 (1.8, 5.3) ^c^	<0.001	1.5 (0.7, 3.0)	2.7 (1.5, 4.5)	<0.001
**Vietnam**, *n*	556	357	777	1299		239	2750	
Cereals and grains	5.2 (3.8, 6.8) ^a^	4.9 (3.9, 6.5) ^a^	5.9 (4.6, 7.5) ^b^	6.3 (4.9, 8.2) ^c^	<0.001	5.1 (3.5, 6.7)	5.9 (4.4, 7.7)	<0.001
Fruits	0.3 (0.0, 1.2)	0.4 (0.0, 1.3)	0.3 (0.0, 1.3)	0.0 (0.0, 1.3)	0.06	0.3 (0.0, 1.1)	0.3 (0.0, 1.3)	0.57
Vegetables	0.4 (0.1, 0.8) ^a^	0.4 (0.1, 0.8) ^a,c^	0.5 (0.2, 0.9) ^b,c^	0.5 (0.1, 1.0) ^b,c^	<0.01	0.4 (0.0, 0.7)	0.5 (0.1, 1.0)	<0.001
Meat and proteinaceous foods	2.3 (1.4, 3.3) ^a^	2.3 (1.5, 3.5) ^a,d^	2.5 (1.6,3.8) ^b,d^	2.7 (1.7, 4.0) ^b,c^	<0.001	2.1 (1.3, 3.3)	2.6 (1.6, 3.8)	<0.001
Extra foods	0.9 (0.3, 1.9) ^a^	1.3 (0.3, 2.2) ^a,c^	1.3 (0.5, 2.5) ^b,c^	1.4 (0.5, 2.7) ^b,c^	<0.001	0.9 (0.3, 2.0)	1.3 (0.5, 2.5)	0.002

Data shown as median interquartile range. Data were analyzed using Kruskal–Wallis tests with post hoc tests (corrected for number of tests). Differences between medians are indicated by different letters.

**Table 4:** Median daily energy and nutrient intakes, including required and estimated average intakes, where applicable, among high-, middle-, low-, and non-dairy consumers as well as among children meeting and not meeting the daily dairy recommendations.

	High-Dairy Consumer	Middle-Dairy Consumer	Low-Dairy Consumer	Non-Dairy Consumer	*p*-Value	Meeting Daily Dairy Recommendations	Not Meeting Daily Dairy Recommendations	*p*-Value
**Indonesia, n**	286	242	337	1351		528	1688	
Energy, kcal	1316 (1039, 1621) ^a^	1189 (915, 1529) ^b^	1125 (838, 1460) ^b,c^	1102 (825, 1414) ^c^	<0.001	1261 (981, 1589)	1108 (827, 1427)	<0.001
Meeting EER, *n* (%)	126 (44.1%) ^+^	59 (24.4%)	68 (20.2%)	193 (14.3%)	<0.001	185 (35.0%)	261 (15.5%)	<0.001
Protein, g	41.8 (34.2, 53.0) ^a^	37.8 (29.8, 48.1) ^b^	34.8 (26.8, 45.6) ^c^	33.5 (23.8, 43.6) ^c^	<0.001	40.4 (31.9, 51.3)	33.8 (24.2, 44.0)	<0.001
Meeting RNI, *n* (%)	266 (93.0%) ^+^	184 (76.0%) ^+^	219 (65.0%) ^+^	590 (43.7%) ^−^	<0.001	450 (85.2%)	809 (47.9%)	<0.001
Carbohydrates, g	167 (137, 214) ^a^	147 (112, 195) ^b^	143 (102, 188) ^b,c^	138 (104, 184) ^c^	<0.001	159 (125, 206)	139 (103, 185)	<0.001
Meeting RNI, *n* (%)	60 (21.0%) ^+^	27 (11.2%)	30 (8.9%)	92 (6.8%) ^−^	<0.001	87 (16.5%)	122 (7.2%)	<0.001
Fat, g	50.3 (37.3, 65.9) ^a^	47 (33.9, 60.3) ^a,b^	44.5 (33.1, 60.8) ^b^	42.9 (30.2, 58.5) ^b^	<0.001	48.4 (35.8, 64.6)	43.4 (30.9, 59.0)	<0.001
Fiber, g	4.85 (3.13, 7.26) ^a^	4.55 (2.53, 6.59) ^a,b^	4.19 (2.61, 7.05) ^a,b^	3.90 (2.30, 6.49) ^b^	0.001	4.73 (2.80, 7.06)	3.97 (2.34, 6.57)	<0.01
Meeting RNI, *n* (%)	0 (0.0%)	1 (0.4%)	0 (0.0%)	4 (0.3%)	0.63	1 (0.2%)	4 (0.2%)	0.84
Vitamin A, µg RE	672 (511, 970) ^a^	443 (314, 681) ^b^	377 (229, 588) ^c^	263 (129, 502) ^d^	<0.001	585 (394, 865)	288 (145, 529)	<0.001
Meeting RNI, *n* (%)	242 (84.6%) ^+^	122 (50.4%) ^+^	125 (37.1%)	348 (25.8%) ^−^	<0.001	364 (68.9%)	473 (28.0%)	<0.001
Meeting EAR, *n* (%)	276 (96.5%) ^+^	172 (71.1%) ^+^	182 (54.0%)	513 (38.0%) ^−^	<0.001	448 (84.8%)	695 (41.2%)	<0.001
Vitamin B_1_, mg	1.01 (0.72, 1.42) ^a^	0.78 (0.57, 1.01) ^b^	0.59 (0.43, 0.83) ^c^	0.43 (0.27, 0.68) ^d^	<0.001	0.89 (0.65, 1.23)	0.46 (0.29, 0.72)	<0.001
Meeting RNI, *n* (%)	265 (92.7%) ^+^	158 (65.3%) ^+^	129 (38.3%)	270 (20.0%) ^−^	<0.001	423 (80.1%)	399 (23.6%)	<0.001
Meeting EAR, *n* (%)	279 (97.6%) ^+^	186 (76.9%) ^+^	194 (57.6%) ^+^	379 (28.1%) ^−^	<0.001	465 (88.1%)	573 (33.9%)	<0.001
Vitamin B_2_, mg	1.43 (1.09, 1.97) ^a^	1.05 (0.75, 1.41) ^b^	0.83 (0.63, 1.17) ^c^	0.60 (0.40, 0.87) ^d^	<0.001	1.27 (0.94, 1.71)	0.65 (0.43, 0.93)	<0.001
Meeting RNI, *n* (%)	282 (98.6%) ^+^	197 (81.4%) ^+^	225 (66.8%) ^+^	420 (31.1%) ^−^	<0.001	479 (90.7%)	645 (38.2%)	<0.001
Meeting EAR, *n* (%)	284 (99.3%) ^+^	216 (89.3%) ^+^	278 (82.5%) ^+^	587 (43.4%) ^−^	<0.001	500 (94.7%)	865 (51.2%)	<0.001
Vitamin B_12_, µg	3.20 (2.10, 4.51) ^a^	2.78 (1.98, 3.91) ^b^	2.37 (1.53, 3.76) ^c^	1.90 (1.06, 3.19) ^d^	<0.001	2.95 (2.05, 4.34)	2.01 (1.15, 3.29)	<0.001
Meeting RNI, *n* (%)	252 (88.1%) ^+^	173 (71.5%) ^+^	234 (69.4%) ^+^	599 (44.3%) ^−^	<0.001	425 (80.5%)	833 (49.3%)	<0.001
Meeting EAR, *n* (%)	269 (94.1%) ^+^	199 (82.2%) ^+^	256 (76.0%) ^+^	701 (51.9%) ^−^	<0.001	468 (88.6%)	957 (56.7%)	<0.001
Vitamin C, mg	63 (43, 95) ^a^	26 (13, 47) ^b^	14 (5, 30) ^c^	9 (2, 23) ^d^	<0.001	46 (25, 75)	9 (3, 24)	<0.001
Meeting RNI, *n* (%)	219 (76.6%) ^+^	65 (26.9%)	51 (15.1%) ^−^	159 (11.8%) ^−^	<0.001	284 (53.8%)	210 (12.4%)	<0.001
Meeting EAR, *n* (%)	241 (84.3%) ^+^	86 (35.5%) ^+^	71 (21.1%) ^−^	191 (14.1%) ^−^	<0.001	327 (61.9%)	262 (15.5%)	<0.001
Vitamin D, µg	10.1 (7.5, 14.5) ^a^	5.5 (4.2, 8.3) ^b^	4.4 (3.2, 5.5) ^c^	1.3 (0.6, 2.3) ^d^	<0.001	8.1 (5.1, 11.8)	1.6 (0.7, 3.4)	<0.001
Meeting RNI, *n* (%)	65 (22.7%) ^+^	10 (4.1%)	2 (0.6%) ^−^	13 (1.0%) ^−^	<0.001	75 (14.2%)	15 (0.9%)	<0.001
Meeting EAR, *n* (%)	147 (51.4%) ^+^	31 (12.8%)	14 (4.2%) ^−^	22 (1.6%) ^−^	<0.001	178 (33.7%)	36 (2.1%)	<0.001
Calcium, mg	900 (708, 1208) ^a^	609 (456, 862) ^b^	497 (386, 639) ^c^	252 (163, 367) ^d^	<0.001	786 (543, 1060)	283 (186, 442)	<0.001
Meeting RNI, *n* (%)	199 (69.6%) ^+^	62 (25.6%) ^+^	20 (5.9%) ^−^	12 (0.9%) ^−^	<0.001	261 (49.4%)	2 (1.9%)	<0.001
Meeting EAR, *n* (%)	233 (81.5%) ^+^	95 (39.3%) ^+^	56 (16.6%)	20 (1.5%) ^−^	<0.001	328 (62.1%)	76 (4.5%)	<0.001
Iron, mg	11.9 (8.9, 15.1) ^a^	7.5 (5.3, 10.5) ^b^	6.4 (4.3, 8.6) ^c^	6.1 (4.2, 8.5) ^c^	<0.001	9.7 (6.7, 13.2)	6.2 (4.2, 8.5)	<0.001
Meeting RNI, *n* (%)	234 (81.8%) ^+^	117 (48.3%)	116 (34.4%) ^−^	477 (35.3%) ^−^	<0.001	351 (66.5%)	593 (35.1%)	<0.001
Meeting EAR, *n* (%)	278 (97.2%) ^+^	219 (90.5%) ^+^	267 (79.2%)	976 (72.2%) ^−^	<0.001	497 (94.1%)	1243 (73.6%)	<0.001
Zinc, mg	7.91 (6.06, 10.40) ^a^	5.26 (3.68, 6.95) ^b^	4.20 (3.19, 5.69) ^c^	4.26 (3.08, 5.77) ^c^	<0.001	6.77 (4.71, 9.00)	4.24 (3.10, 5.72)	<0.001
Meeting RNI, *n* (%)	266 (93.0%) ^+^	161 (66.5%) ^+^	146 (43.3%)	438 (32.4%) ^−^	<0.001	427 (80.9%)	584 (34.6%)	<0.001
Meeting EAR, *n* (%)	277 (96.9%) ^+^	181 (74.8%) ^+^	184 (54.6%)	580 (42.9%) ^−^	<0.001	458 (86.7%)	764 (45.3%)	<0.001
Choline, mg	239 (140, 314) ^a^	197 (104, 288) ^b^	205 (104, 311) ^a,b^	190 (97, 278) ^b^	<0.001	215 (125, 300)	192 (98, 284)	<0.001
Meeting RNI, *n* (%)	157 (54.9%) ^+^	77 (31.8%)	108 (32.0%)	247 (18.3%) ^−^	<0.001	234 (44.3%)	355 (21.0%)	<0.001
DHA, mg	53.1 (29.9, 88.1) ^a^	32.6 (18.2, 71.3) ^b^	28.5 (16.3, 59.0) ^b^	29.1 (11.8, 62.0) ^b^	<0.001	42.9 (23.4, 82.4)	29 (12.7, 61.1)	<0.05
**Malaysia, n**	417	423	538	1070		423	2025	
Energy, kcal	1415 (1174, 1724) ^a^	1373 (1137, 1755) ^a,b^	1386 (1113, 1697) ^a,b^	1342 (1055, 1658) ^b^	<0.01	1420 (1174, 1737)	1361 (1091, 1688)	<0.01
Meeting EER, *n* (%)	328 (78.7%) ^+^	209 (49.4%)	203 (37.7%) ^−^	329 (30.7%) ^−^	<0.001	333 (78.7%)	736 (36.3%)	<0.001
Protein, g	51.4 (40.9, 63.1) ^a^	53.3 (40.7, 67.2) ^a^	50.3 (37.7, 65.2) ^a,b^	48.2 (36.5, 63.3) ^b^	<0.001	51.7 (40.9, 63.3)	49.7 (37.5, 64.4)	0.14
Meeting RNI, *n* (%)	417 (100.0%)	420 (99.3%)	517 (96.1%)	1006 (94.0%)	<0.001	423.0 (100.0%)	1937.0 (95.7%)	<0.001
Carbohydrates, g	193 (158, 235) ^a^	181 (153, 236) ^a,b^	186 (146, 239) ^a,b^	184 (140, 231) ^b^	<0.05	193 (158, 236)	183 (145, 235)	<0.01
Fat, g	47.6 (38.8, 60.3) ^a^	48.3 (35.8, 62.4) ^a^	46.9 (34.3, 61.0) ^a^	44.2 (32.2, 59.3) ^b^	<0.001	47.8 (38.8, 60.6)	45.7 (33.5, 60.7)	<0.05
Meeting RNI, *n* (%)	357 (85.6%) ^+^	310 (73.3%) ^+^	324 (60.2%)	536 (50.1%) ^−^	<0.001	362.0 (85.6%)	1165.0 (57.5%)	<0.001
Vitamin A, µg RE	816 (616, 1062) ^a^	714 (502, 1024) ^b^	639 (436, 955) ^c^	510 (316, 762) ^d^	<0.001	816 (619, 1066)	585 (385, 881)	<0.001
Meeting RNI, *n* (%)	390 (93.5%) ^+^	340 (80.4%) ^+^	370 (68.8%)	522 (48.8%) ^−^	<0.001	396.0 (93.6%)	1226.0 (60.5%)	<0.001
Meeting EAR, *n* (%)	412 (98.8%) ^+^	392 (92.7%) ^+^	457 (84.9%)	738 (69.0%) ^−^	<0.001	418.0 (98.8%)	1581.0 (78.1%)	<0.001
β-carotene, µg	730 (344, 1414) ^a^	800 (322, 1994) ^a^	807 (237, 1882) ^a^	610 (192, 1460) ^b^	<0.001	724 (339, 1414)	664 (229, 1660)	0.14
Vitamin B_1_, mg	1.27 (0.93, 1.77) ^a^	1.23 (0.85, 1.69) ^a^	1.07 (0.71, 1.48) ^b^	0.79 (0.51, 1.15) ^c^	<0.001	1.28 (0.93, 1.77)	0.95 (0.62, 1.35)	<0.001
Meeting RNI, *n* (%)	401 (96.2%) ^+^	364 (86.1%) ^+^	383 (71.2%)	447 (41.8%) ^−^	<0.001	407.0 (96.2%)	1188.0 (58.7%)	<0.001
Meeting EAR, *n* (%)	410 (98.3%) ^+^	388 (91.7%) ^+^	432 (80.3%)	609 (56.9%) ^−^	<0.001	416.0 (98.3%)	1423.0 (70.3%)	<0.001
Vitamin B_2_, mg	1.74 (1.30, 2.25) ^a^	1.55 (1.15, 1.95) ^b^	1.31 (0.97, 1.78) ^c^	1.07 (0.75, 1.45) ^d^	<0.001	1.74 (1.31, 2.27)	1.23 (0.86, 1.69)	<0.001
Meeting RNI, *n* (%)	415 (99.5%) ^+^	403 (95.3%) ^+^	441 (82.0%)	665 (62.1%) ^−^	<0.001	421.0 (99.5%)	1503.0 (74.2%)	<0.001
Meeting EAR, *n* (%)	416 (99.8%) ^+^	415 (98.1%) ^+^	501 (93.1%)	829 (77.5%) ^−^	<0.001	422.0 (99.8%)	1739.0 (85.9%)	<0.001
Vitamin B_3_, mg	12.7 (9.8, 16.7) ^a^	11.7 (8.9, 15.9) ^a,b^	11.5 (8.0, 15.5) ^b^	9.5 (6.5, 13.5) ^c^	<0.001	12.7 (9.8, 16.8)	10.5 (7.4, 14.5)	<0.001
Meeting RNI, *n* (%)	374 (89.7%) ^+^	283 (66.9%) ^+^	291 (54.1%)	358 (33.5%) ^−^	<0.001	379 (89.6%)	927 (45.8%)	<0.001
Meeting EAR, *n* (%)	401 (96.2%) ^+^	359 (84.9%) ^+^	394 (73.2%)	574 (53.6%) ^−^	<0.001	407 (96.2%)	1321 (65.2%)	<0.001
Vitamin B_12_, µg	3.10 (2.18, 4.44) ^a^	2.38 (1.49, 3.79) ^b^	2.14 (1.29, 3.74) ^b,c^	2.00 (1.00, 3.76) ^c^	<0.001	3.10 (2.18, 4.44)	2.16 (1.16, 3.74)	<0.001
Meeting RNI, *n* (%)	368 (88.2%) ^+^	249 (58.9%)	254 (47.2%) ^−^	457 (42.7%) ^−^	<0.001	372.0 (87.9%)	956.0 (47.2%)	<0.001
Vitamin C, mg	109 (69, 159) ^a^	63 (41, 111) ^b^	51 (30, 93) ^c^	37 (19, 71) ^d^	<0.001	109 (68, 159)	46 (25, 87)	<0.001
Meeting RNI, *n* (%)	384 (92.1%) ^+^	289 (68.3%) ^+^	265 (49.3%)	344 (32.1%) ^−^	<0.001	387.0 (91.5%)	895.0 (44.2%)	<0.001
Meeting EAR, *n* (%)	407 (97.6%) ^+^	357 (84.4%) ^+^	392 (72.9%)	553 (51.7%) ^−^	<0.001	410.0 (96.9%)	1299.0 (64.1%)	<0.001
Vitamin D, µg	10.3 (7.5, 14.2) ^a^	6.3 (4.0, 8.6) ^b^	4.3 (2.5, 6.4) ^c^	2.4 (1.1, 4.1) ^d^	<0.001	10.3 (7.4, 14.2)	3.5 (1.8, 6.0)	<0.001
Meeting RNI, *n* (%)	84 (20.1%) ^+^	9 (2.1%) ^−^	6 (1.1%) ^−^	5 (0.5%) ^−^	<0.001	84.0 (19.9%)	20.0 (1.0%)	<0.001
Meeting EAR, *n* (%)	221 (53.0%) ^+^	70 (16.5%)	44 (8.2%) ^−^	36 (3.4%) ^−^	<0.001	223.0 (52.7%)	148.0 (7.3%)	<0.001
Calcium, mg	989 (792, 1311) ^a^	707 (560, 892) ^b^	562 (428, 729) ^c^	383 (257, 549) ^d^	<0.001	988 (792, 1316)	498 (334, 689)	<0.001
Meeting RNI, *n* (%)	268 (64.3%) ^+^	61 (14.4%)	28 (5.2%) ^−^	18 (1.7%) ^−^	<0.001	270.0 (63.8%)	105.0 (5.2%)	<0.001
Meeting EAR, *n* (%)	359 (86.1%) ^+^	164 (38.8%) ^+^	81 (15.1%) ^−^	49 (4.6%) ^−^	<0.001	363.0 (85.8%)	290.0 (14.3%)	<0.001
Iron, mg	14.4 (11.2, 17.9) ^a^	11.6 (9.1, 15.1) ^b^	11 (8.1, 14.7) ^b^	9.4 (6.8, 13.1) ^c^	<0.001	14.4 (11.2, 18.0)	10.3 (7.6, 14.1)	<0.001
Meeting RNI, *n* (%)	411 (98.6%) ^+^	381 (90.3%) ^+^	451 (84.1%)	703 (66.1%) ^−^	<0.001	416.0 (98.3%)	1530.0 (75.9%)	<0.001
Meeting EAR, *n* (%	412 (98.8%) ^+^	394 (93.4%)	475 (88.6%)	809 (76.0%) ^−^	<0.001	418.0 (98.8%)	1672.0 (82.9%)	<0.001
Sodium, mg	1454 (1026, 1911) ^a^	1648 (1179, 2350) ^b^	1727 (1218, 2355) ^b^	1655 (1151, 2423) ^b^	<0.001	1463 (1026, 1916)	1674 (1178, 2379)	<0.001
Meeting RNI, *n* (%)	288 (69.1%)	315 (74.5%)	389 (72.3%)	701 (65.5%)	<0.01	293.0 (69.3%)	1400.0 (69.1%)	0.96
Potassium, mg	1425 (1113, 1803) ^a^	1134 (899, 1431) ^b^	1007 (777, 1301) ^c^	855 (628, 1178) ^d^	<0.001	1425 (1113, 1803)	958 (715, 1271)	<0.001
Meeting RNI, *n* (%)	1 (0.2%)	1 (0.2%)	0 (0.0%)	0 (0.0%)	0.17	1.0 (0.2%)	1.0 (0.0%)	0.32
Phosphorus, mg	889 (724, 1117) ^a^	771 (599, 968) ^b^	661 (499, 867) ^c^	565 (408, 757) ^d^	<0.001	893 (730, 1120)	633 (466, 847)	<0.001
Meeting RNI, *n* (%)	397 (95.2%) ^+^	276 (65.2%) ^+^	250 (46.5%)	288 (26.9%) ^−^	<0.001	401.0 (94.8%)	810.0 (40.0%)	<0.001
**Thailand,** **n**	619	527	796	704		620	2026	
Energy, kcal	1446 (1195, 1724) ^a^	1421 (1114, 1794) ^a^	1501 (1202, 1872) ^b^	1463 (1177, 1870) ^a,b^	<0.01	1446 (1197, 1724)	1468 (1178, 1862)	0.11
Meeting EER, *n* (%)	500 (80.8%) ^+^	346 (65.7%)	462 (58.0%)	356 (50.6%) ^−^	<0.001	501 (80.8%)	1163 (57.4%)	<0.001
Protein, g	57 (47.0, 71.5) ^a^	53.9 (43.2, 69.9) ^b^	56.1 (43.1, 71.9) ^a,b^	51.7 (38.3, 67.1) ^c^	<0.001	57 (47.1, 71.5)	53.7 (41.8, 69.9)	<0.001
Meeting RNI, *n* (%)	616 (99.5%)	525 (99.6%)	777 (97.6%)	635 (90.2%)	<0.001	617 (99.5%)	1936 (95.6%)	<0.001
Meeting EAR, *n* (%)	618 (99.8%)	527 (100.0%)	793 (99.6%)	686 (97.4%)	<0.001	619 (99.8%)	2005 (99.0%)	<0.05
Carbohydrates, g	168 (127, 206) ^a^	177 (135, 228) ^b^	197 (154, 248) ^c^	201 (158, 260) ^c^	<0.001	168 (127, 205)	193 (150, 246)	<0.001
Fat, g	58.5 (45.9, 73.5) ^a^	52.2 (39.0, 68.6) ^b^	52.5 (38.5, 67.5) ^b^	47 (31.8, 64.6) ^c^	<0.001	58.5 (45.9, 73.4)	50.5 (36.7, 66.8)	<0.001
Fiber, g	6.16 (3.70, 8.49) ^a^	5.77 (4.13, 7.95) ^a^	5.76 (4.16, 8.05) ^a^	5.27 (3.55, 7.53) ^b^	<0.001	6.17 (3.71, 8.50)	5.56 (3.91, 7.88)	0.11
Meeting RNI, *n* (%)	161 (26.0%) ^+^	67 (12.7%)	53 (6.7%) ^−^	39 (5.5%) ^−^	<0.001	161 (26.0%)	159 (7.8%)	<0.001
Vitamin A, µg RAE	498 (360, 701) ^a^	373 (251, 534) ^b^	281 (175, 422) ^c^	218 (117, 376) ^d^	<0.001	498 (360, 694)	281 (170, 441)	<0.001
Meeting RNI, *n* (%)	498 (80.5%) ^+^	275 (52.2%) ^+^	254 (31.9%) ^−^	147 (20.9%) ^−^	<0.001	498 (80.3%)	676 (33.4%)	<0.001
Meeting EAR, *n* (%)	535 (86.4%) ^+^	331 (62.8%) ^+^	307 (38.6%) ^−^	199 (28.3%) ^−^	<0.001	535 (86.3%)	837 (41.3%)	<0.001
Vitamin B_1_, mg	0.86 (0.58, 1.36) ^a^	0.91 (0.59, 1.34) ^a^	0.81 (0.55, 1.25) ^a^	0.75 (0.47, 1.18) ^b^	<0.001	0.87 (0.58, 1.35)	0.82 (0.54, 1.26)	<0.01
Meeting RNI, *n* (%)	547 (88.4%) ^+^	447 (84.8%) ^+^	599 (75.3%)	460 (65.3%) ^−^	<0.001	548 (88.4%)	1505 (74.3%)	<0.001
Meeting EAR, *n* (%)	502 (81.1%) ^+^	395 (75.0%)	503 (63.2%)	366 (52.0%) ^−^	<0.001	503 (81.1%)	1263 (62.3%)	<0.001
Vitamin B_2_, mg	1.81 (1.49, 2.21) ^a^	1.36 (1.11, 1.70) ^b^	1.10 (0.86, 1.45) ^c^	0.79 (0.54, 1.15) ^d^	<0.001	1.81 (1.49, 2.21)	1.09 (0.79, 1.47)	<0.001
Meeting RNI, *n* (%)	619 (100.0%) ^+^	515 (97.7%) ^+^	712 (89.4%)	382 (54.3%) ^−^	<0.001	620 (100.0%)	1608 (79.4%)	<0.001
Meeting EAR, *n* (%)	619 (100.0%) ^+^	524 (99.4%) ^+^	762 (95.7%)	456 (64.8%) ^−^	<0.001	620 (100.0%)	1741 (85.9%)	<0.001
Vitamin B_3_, mg	8.5 (5.4, 12.7) ^a^	9.5 (6.2, 13.9) ^b^	10.4 (7.3, 14.7) ^c^	10 (6.9, 13.9) ^b,c^	<0.001	8.5 (5.5, 12.7)	10.1 (6.9, 14.3)	<0.001
Meeting RNI, *n* (%)	384 (62.0%)	334(63.4%)	490 (61.6%)	386 (54.8%)	0.007	385 (62.1%)	1209 (59.7%)	0.28
Meeting EAR, *n* (%)	457 (73.8%)	416 (78.9%)	633 (79.5%)	496 (70.5%)	<0.001	458 (73.9%)	1544 (76.2%)	0.24
Vitamin B_12_, µg	2.71 (1.59, 4.11) ^a^	2.21 (1.42, 3.07) ^b^	1.92 (1.31, 2.85) ^c^	1.49 (0.83, 2.46) ^d^	<0.001	2.70 (1.58, 4.11)	1.84 (1.14, 2.82)	<0.001
Meeting RNI, *n* (%)	497 (80.3%) ^+^	394 (74.8%) ^+^	473 (59.4%)	302 (42.9%) ^−^	<0.001	497 (80.2%)	1169 (57.7%)	<0.001
Meeting EAR, *n* (%)	549 (88.7%) ^+^	463 (87.9%) ^+^	640 (80.4%)	427 (60.7%) ^−^	<0.001	550 (88.7%)	1529 (75.5%)	<0.001
Vitamin C, mg	21 (9, 48)	21 (10, 35)	22 (11, 38)	21 (9, 41)	0.48	21 (9, 48)	22 (10, 38)	0.36
Meeting RNI, *n* (%)	244 (39.4%) ^+^	153 (29.0%)	204 (25.6%)^−^	177 (25.1%) ^−^	<0.001	245 (39.5%)	533 (26.3%)	<0.001
Meeting EAR, *n* (%)	286 (46.2%) ^+^	201 (38.1%)	257 (32.3%)	205 (29.1%) ^−^	<0.001	287 (46.3%)	662 (32.7%)	<0.001
Vitamin D, µg	7.6 (5.7, 9.8) ^a^	5.3 (3.9, 6.9) ^b^	3.7 (2.6, 5.4) ^c^	2.1 (1.0, 3.8) ^d^	<0.001	7.6 (5.6, 9.8)	3.7 (2.2, 5.5)	<0.001
Meeting RNI, *n* (%)	25 (4.0%) ^+^	8 (1.5%)	6 (0.8%) ^−^	5 (0.7%)	<0.001	25 (4.0%)	19 (0.9%)	<0.001
Meeting EAR, *n* (%)	144 (23.3%) ^+^	29 (5.5%) ^−^	29 (3.6%) ^−^	12 (1.7%) ^−^	<0.001	144 (23.2%)	70 (3.5%)	<0.001
Calcium, mg	836 (688, 1048) ^a^	553 (475, 669) ^b^	398 (314, 514) ^c^	211 (125, 347) ^d^	<0.001	835 (689, 1048)	397 (261, 543)	<0.001
Meeting RNI, *n* (%)	479 (77.4%) ^+^	125 (23.7%)	37 (4.6%) ^−^	23 (3.3%) ^−^	<0.001	479 (77.3%)	185 (9.1%)	<0.001
Iron, mg	5.9 (3.9, 10.5)	5.9 (4.3, 8.4)	6.4 (4.7, 8.9)	6.3 (4.6, 8.7)	0.06	5.9 (3.9, 10.5)	6.3 (4.5, 8.7)	0.52
Meeting RNI, *n* (%)	317 (51.2%) ^+^	241 (45.7%)	344 (43.2%)	250 (35.5%) ^−^	<0.001	317 (51.1%)	835 (41.2%)	<0.001
Meeting EAR, *n* (%)	367 (59.3%) ^+^	300 (56.9%)	411 (51.6%)	300 (42.6%) ^−^	<0.001	368 (59.4%)	1010 (49.9%)	<0.001
Zinc, mg	4.01 (2.89, 5.83) ^a,b^	3.85 (2.90, 5.14) ^a^	4.18 (3.18, 5.56) ^b^	4.17 (2.99, 5.58) ^a,b^	<0.05	4.01 (2.89, 5.82)	4.10 (3.06, 5.47)	0.55
Meeting RNI, *n* (%)	209 (33.8%) ^+^	98 (18.6%)	111 (13.9%) ^−^	89 (12.6%) ^−^	<0.001	209 (33.7%)	298 (14.7%)	<0.001
Meeting EAR, *n* (%)	274 (44.3%) ^+^	154 (29.2%)	212 (26.6%)	147 (20.9%) ^−^	<0.001	274 (44.2%)	513 (25.3%)	<0.001
Magnesium, mg	81.3 (51.5, 110.0) ^a^	83.5 (60.5, 115.4) ^a,c^	96.6 (69.7, 128.9) ^b^	92.5(62.5, 129.8) ^b,c^	<0.001	81.3 (51.8, 110.2)	91.8 (64.8, 126.7)	<0.001
Meeting RNI, *n* (%)	331 (53.5%) ^+^	234 (44.4%)	337 (42.3%)	203 (28.8%) ^−^	<0.001	332 (53.5%)	773 (38.2%)	<0.001
Meeting EAR, *n* (%)	412 (66.6%) ^+^	316 (60.0%)	416 (52.3%)	288 (40.9%) ^−^	<0.001	413 (66.6%)	1019 (50.3%)	<0.001
Sodium, mg	1748 (1182, 2357) ^a^	1920 (1344, 2668) ^b^	2061 (1494, 2882) ^c^	2109 (1438, 3023) ^c^	<0.001	1751 (1183, 2352)	2038 (1429, 2860)	<0.001
Meeting RNI, *n* (%)	613 (99.0%)	517 (98.1%)	787 (98.9%)	688 (97.7%)	0.16	614 (99.0%)	1991 (98.3%)	0.18
Potassium, mg	1188 (798, 1634) ^a^	1014 (768, 1387) ^b,c^	1071 (795, 1415) ^b^	981 (710, 1355) ^c^	<0.001	1186 (799, 1628)	1036 (753, 1389)	<0.001
Meeting RNI, *n* (%)	122 (19.7%) ^+^	21 (4.0%) ^−^	16 (2.0%) ^−^	19 (2.7%) ^−^	<0.001	122 (19.7%)	56 (2.8%)	<0.001
Phosphorus, mg	702 (480, 929) ^a^	595 (443, 813) ^b^	615 (455, 807) ^b^	518 (377, 709) ^c^	<0.001	703 (481, 928)	569 (420, 784))	<0.001
Meeting RNI, *n* (%)	442 (71.4%) ^+^	307 (58.3%) ^+^	392 (49.2%)	224 (31.8%) ^−^	<0.001	443 (71.5%)	922 (45.5%)	<0.001
Meeting EAR, *n* (%)	504 (81.4%) ^+^	392 (74.4%) ^+^	511 (64.2%)	318 (45.2%) ^−^	<0.001	505 (81.5%)	1220 (60.2%)	<0.001
**Vietnam,** **n**	556	357	777	1299		239	2750	
Energy, kcal	1196 (953, 1459) ^a^	1017 (836, 1317) ^b^	1120 (887, 1395) ^c^	1087 (845, 1398) ^b,c^	<0.001	1233 (997, 1506)	1092 (856, 1391)	<0.001
Meeting EER, *n* (%)	234 (42.1%) ^+^	70 (19.6%)	118 (15.2%) ^−^	142 (10.9%) ^−^	<0.001	125 (52.3%)	439 (16.0%)	<0.001
Protein, g	51.2 (40.0, 65.1) ^a^	45.4 (35.1, 59.2) ^b,c^	47.3 (36.3, 62.7) ^b^	45.5 (33.5, 60.4) ^c^	<0.001	52.5 (40.5, 68.7)	46.7 (35.2, 61.0)	<0.001
Meeting RNI, *n* (%)	530 (95.3%) ^+^	325 (91.0%)	661 (85.1%)	927 (71.4%) ^−^	<0.001	231 (96.7%)	2212 (80.4%)	<0.001
Carbohydrates, g	166 (129, 202) ^a^	148 (116, 190) ^b^	165 (132, 206) ^a,c^	172 (133, 219) ^c^	<0.001	167 (133, 205)	165 (129, 209)	0.38
Meeting RNI, *n* (%)	227 (40.8%) ^+^	92 (25.8%)	181 (23.3%)	298 (22.9%) ^−^	<0.001	124 (51.9%)	674 (24.5%)	<0.001
Fat, g	33.5 (25.9, 43.7) ^a^	27.6 (20.2, 36.3) ^b^	26.8 (18.7, 37.5) ^b^	21.5 (13.9, 32.5) ^c^	<0.001	36.4 (27.9, 46.3)	25.2 (17.2, 35.9)	<0.001
Meeting RNI, *n* (%)	259 (46.6%) ^+^	100 (28.0%)	206 (26.5%)	220 (16.9%) ^−^	<0.001	133 (55.6%)	652 (23.7%)	<0.001
Vitamin A, µg RAE	419 (311, 575) ^a^	304 (213, 477) ^b^	245 (157, 436) ^c^	215 (84, 476) ^d^	<0.001	477 (385, 654)	266 (146, 468)	<0.001
Meeting RNI, *n* (%)	259 (46.6%) ^+^	104 (29.1%)	184 (23.7%) ^−^	314 (24.2%) ^−^	<0.001	144 (60.3%)	717 (26.1%)	<0.001
Meeting EAR, *n* (%)	407 (73.2%) ^+^	177 (49.6%)	284 (36.6%) ^−^	429 (33.0%) ^−^	<0.001	207 (86.6%)	1090 (39.6%)	<0.001
Vitamin B_12_, µg	2.58 (1.94, 3.60) ^a^	1.73 (1.30, 2.61) ^b^	1.49 (1.01, 2.34) ^c^	1.00 (0.48, 2.00) ^d^	<0.001	2.92 (2.25, 4.05)	1.47 (0.83, 2.40)	<0.001
Meeting RNI, *n* (%)	550 (98.9%) ^+^	305 (85.4%) ^+^	488 (62.8%)	501 (38.6%) ^−^	<0.001	239 (100.0%)	1605 (58.4%)	<0.001
Meeting EAR, *n* (%)	551 (99.1%) ^+^	320 (89.6%) ^+^	562 (72.3%)	576 (44.3%) ^−^	<0.001	239 (100.0%)	1770 (64.4%)	<0.001
Vitamin C, mg	28 (11, 55) ^a^	28 (16, 58) ^ab^	25 (12, 52) ^a,b^	21 (7, 49) ^b^	<0.001	29 (11, 63)	24 (10, 51)	<0.01
Meeting RNI, *n* (%)	186 (33.5%) ^+^	114 (31.9%) ^+^	200 (25.7%)	288 (22.2%) ^−^	<0.001	88 (36.8%)	700 (25.5%)	<0.001
Meeting EAR, *n* (%)	223 (40.1%) ^+^	139 (38.9%) ^+^	237 (30.5%)	334 (25.7%) ^−^	<0.001	98 (41.0%)	835 (30.4%)	<0.001
Vitamin D, µg	6.2 (4.3, 12.4) ^a^	3.4 (2.6, 6.6) ^b^	2.5 (1.6, 5.1) ^c^	0.9 (0.4, 2.5) ^d^	<0.001	7.7 (5.5, 15.0)	2.3 (0.8, 4.9)	<0.001
Meeting RNI, *n* (%)	96 (17.3%) ^+^	17 (4.8%)	27 (3.5%) ^−^	54 (4.2%) ^−^	<0.001	60 (25.1%)	134 (4.9%)	<0.001
Calcium, mg	637 (546, 780) ^a^	439 (386, 535) ^b^	366 (299, 458) ^c^	207 (150, 296) ^d^	<0.001	769 (655, 918)	329 (206, 468)	<0.001
Meeting RNI, *n* (%)	330 (59.4%) ^+^	51 (14.3%)	37 (4.8%) ^−^	29 (2.2%) ^−^	<0.001	215 (90.0%)	232 (8.4%)	<0.001
Iron, mg	6.7 (4.8, 9.0) ^a,b^	6.5 (4.7, 8.1) ^a^	6.6 (5.0, 8.6) ^a,b^	6.8 (5.0, 8.9) ^b^	<0.05	6.9 (4.8, 9.8)	6.7 (5.0, 8.6)	0.29
Meeting RNI, *n* (%)	330 (59.4%) ^+^	208 (58.3%)	411 (52.9%)	641 (49.3%) ^−^	<0.001	154 (64.4%)	1436 (52.2%)	<0.001
Zinc, mg	6.73 (5.23, 8.36) ^a^	5.95 (4.65, 7.26) ^b^	6.08 (4.67, 7.73) ^b^	5.84 (4.46, 7.72) ^b^	<0.001	7.15 (5.61, 8.76)	6.02 (4.61, 7.73)	<0.001
Meeting RNI, *n* (%)	447 (80.4%) ^+^	239 (66.9%)	479 (61.6%) ^−^	649 (50.0%) ^−^	<0.001	209 (87.4%)	1605 (58.4%)	<0.001

Data are presented as medians (IQR), frequencies and proportions (%). Data presented as medians (IQR) across the different dairy-consuming groups were analyzed using Kruskal–Wallis tests with post hoc tests (corrected for number of tests). Differences between medians are indicated by different letters. Data presented as medians (IQR) across groups for meeting versus not meeting daily dairy recommendations were analyzed using Wilcoxon’s rank-sum tests. Data presented as frequencies and proportions were analyzed using chi-square tests. ^+^ significantly more likely to meet recommendations/requirements based on standardized residuals > 1.96. ^−^−significantly less likely to meet recommendations/requirements based on standardized residuals < −1.96. EAR, estimated adequate requirement; EER, estimated energy requirements; RNI, recommended nutrient intake.

## Data Availability

The datasets presented in this article are not readily available because they are proprietary and managed by FrieslandCampina, which does not permit external access to the data. Requests to access the datasets should be directed to (cecile.singh-povel@frieslandcampina.com).

## Supplementary Materials

The following supporting information can be downloaded at https://www.mdpi.com/article/10.3390/nu17233740/s1, Table S1: Food intake, among high-, middle-, low-, and non-dairy consumers as well as among children meeting and not meeting the daily dairy recommendations, stratified by age; Table S2: Daily energy and nutrient intakes, including required and estimated average intakes, where applicable, among high-, middle-, low-, and non-dairy consumers as well as among children meeting and not meeting the daily dairy recommendations, stratified by age groups.

## Abbreviations

DHA	Docosahexaenoic Acid
EAR	Estimated Adequate Requirement
EER	Estimated Energy Requirements
IFT	Infant Formula and Toddler
IQR	Interquartile Range
RNI	Recommended Nutrient Intake
SD	Standard Deviation
SEA	Southeast Asia
SEANUTS	South East Asian Nutrition Surveys
YCF	Young Child Formula
