# Supplementary material for: Associations Between Dairy Consumption and Nutrient Intake in Southeast Asian Children: Findings from the South East Asian Nutrition Surveys II (SEANUTS II)
Source: Nutrients. 2025 Nov 28;17(23):3740. doi: 10.3390/nu17233740 (PMC12694304; doi:10.3390/nu17233740)
Supplement: Supplementary file 1 [file nutrients-17-03740-s001.zip › supplementary table S2.pdf]

Supplementary Table S2. Daily median energy and nutrient intakes, including required and estimated average intakes where applicable, among high, middle, low, and non-dairy consumers, as well as among children meeting and not meeting the daily dairy recommendations stratified for age groups.

	**High dairy consumer**	**Middle dairy consumer**	**Low dairy consumer**	**No dairy consumer**	***p*-Value**	**Meeting daily dairy recommendations**	**Not meeting daily dairy recommendations**	***p*-Value**
**2-3 years**								
**Indonesia, n**	170	111	116	232		281	348	
Energy, kcal	1,215.4 (965.7, 1,507.9)	985.8 (780.1, 1,266.7)	932.5 (677.0, 1,201.0)	835.2 (624.3, 1,114.9)	<0.001	1,128.4 (858.7, 1,424.6)	869.9 (653.2, 1,156.9)	<0.001
Meeting EER, n (%)	65 (38.2%)	19 (17.1%)	21 (18.1%)	27 (11.6%)	<0.001	84 (29.9%)	48 (13.8%)	<0.001
Protein, g	38.8 (30.2, 47.2)	31.8 (24.5, 43.2)	29.1 (20.5, 37.3)	24.8 (17.3, 33.7)	<0.001	35.8 (27.7, 45.4)	26.4 (18.6, 35.4)	<0.001
Meeting RNI, n (%)	159 (93.5%)	95 (85.6%)	93 (80.2%)	151 (65.1%)	<0.001	254 (90.4%)	244 (70.1%)	<0.001
Carbohydrates, g	158 (125.5, 193.6)	129.2 (101.0, 163.7)	115.8 (84.9, 156.6)	109.7 (86.9, 150.4)	<0.001	148.4 (112.7, 180.0)	112.8 (86.1, 155.0)	<0.001
Meeting RNI, n (%)	31 (18.2%)	9 (8.1%)	7 (6.0%)	7 (3.0%)	<0.001	40 (14.2%)	14 (4.0%)	<0.001
Fat, g	45.6 (33.7, 59.2)	39 (26.8, 50.3)	39.3 (25.6, 49.1)	31.4 (22.0, 47.5)	<0.001	43.6 (31.2, 54.5)	34 (22.4, 47.5)	<0.001
Fiber, g	4.87 (3.47, 7.18)	3.57 (2.28, 5.79)	3.61 (2.38, 5.46)	2.90 (1.66, 5.03)	<0.001	4.56 (2.78, 6.57)	3.20 (1.84, 5.12)	<0.001
Meeting RNI, n (%)	0 (0.0%)	0 (0.0%)	0 (0.0%)	1 (0.4%)	>0.99	0 (0.0%)	1 (0.3%)	>0.99
Vitamin A, μg RE	661.6 (505.7, 956.1)	441.6 (308.0, 729.8)	378.9 (242.8, 584.7)	283.9 (140.2, 527.1)	<0.001	584.3 (417.2, 869.0)	318.2 (167.8, 546.1)	<0.001
Meeting RNI, n (%)	149 (87.6%)	63 (56.8%)	53 (45.7%)	83 (35.8%)	<0.001	212 (75.4%)	136 (39.1%)	<0.001
Meeting EAR, n (%)	168 (98.8%)	88 (79.3%)	73 (62.9%)	113 (48.7%)	<0.001	256 (91.1%)	186 (53.4%)	<0.001
Vitamin B_1, mg	0.85 (0.64, 1.16)	0.68 (0.48, 0.97)	0.51 (0.38, 0.74)	0.39 (0.21, 0.66)	<0.001	0.79 (0.59, 1.07)	0.46 (0.26, 0.69)	<0.001
Meeting RNI, n (%)	155 (91.2%)	81 (73.0%)	59 (50.9%)	90 (38.8%)	<0.001	236 (84.0%)	149 (42.8%)	<0.001
Meeting EAR, n (%)	164 (96.5%)	90 (81.1%)	82 (70.7%)	114 (49.1%)	<0.001	254 (90.4%)	196 (56.3%)	<0.001
Vitamin B_2, mg	1.30 (1.00, 1.69)	0.87 (0.67, 1.26)	0.70 (0.52, 0.95)	1.30 (1.00, 1.69)	<0.001	1.11 (0.81, 1.50)	0.57 (0.37, 0.86)	<0.001
Meeting RNI, n (%)	168 (98.8%)	100 (90.1%)	92 (79.3%)	118 (50.9%)	<0.001	268 (95.4%)	210 (60.3%)	<0.001
Meeting EAR, n (%)	169 (99.4%)	107 (96.4%)	104 (89.7%)	145 (62.5%)	<0.001	276 (98.2%)	249 (71.6%)	<0.001
Vitamin B_{12}, μg	2.61 (1.86, 4.11)	2.56 (1.48, 3.89)	2.11 (1.19, 3.12)	1.57 (0.75, 2.85)	<0.001	2.58 (1.75, 3.93)	1.73 (0.96, 3.00)	<0.001
Meeting RNI, n (%)	144 (84.7%)	83 (74.8%)	80 (69.0%)	123 (53.0%)	<0.001	227 (80.8%)	203 (58.3%)	<0.001
Meeting EAR, n (%)	158 (92.9%)	95 (85.6%)	87 (75.0%)	145 (62.5%)	<0.001	253 (90.0%)	232 (66.7%)	<0.001

Vitamin C, mg	62.8 (44.2, 99.2)	23.5 (11.3, 37.0)	13.7 (5.3, 32.9)	8.4 (2.8, 25.2)	<0.001	46.7 (27.4, 74.5)	10.3 (3.6, 27.4)	<0.001
Meeting RNI, n (%)	138 (81.2%)	24 (21.6%)	17 (14.7%)	32 (13.8%)	<0.001	162 (57.7%)	49 (14.1%)	<0.001
Meeting EAR, n (%)	153 (90.0%)	37 (33.3%)	29 (25.0%)	44 (19.0%)	<0.001	190 (67.6%)	73 (21.0%)	<0.001
Vitamin D, μg	9.7 (6.8, 13.6)	5.5 (4.0, 8.1)	4.1 (2.9, 5.5)	1.1 (0.4, 2.1)	<0.001	8 (5.3, 11.6)	1.9 (0.7, 3.8)	<0.001
Meeting RNI, n (%)	37 (21.8%)	7 (6.3%)	1 (0.9%)	2 (0.9%)	<0.001	44 (15.7%)	3 (0.9%)	<0.001
Meeting EAR, n (%)	82 (48.2%)	15 (13.5%)	6 (5.2%)	3 (1.3%)	<0.001	97 (34.5%)	9 (2.6%)	<0.001
Calcium, mg	810.7 (657.5, 1,062.0)	574.1 (416.4, 861.7)	479.7 (325.4, 592.7)	226.4 (138.6, 314.3)	<0.001	752.6 (537.0, 1,013.0)	284 (171.1, 440.6)	<0.001
Meeting RNI, n (%)	131 (77.1%)	45 (40.5%)	17 (14.7%)	3 (1.3%)	<0.001	176 (62.6%)	20 (5.7%)	<0.001
Meeting EAR, n (%)	147 (86.5%)	59 (53.2%)	39 (33.6%)	7 (3.0%)	<0.001	206 (73.3%)	46 (13.2%)	<0.001
Iron, mg	10.5 (8.1, 13.4)	6.2 (4.6, 8.2)	5.5 (3.4, 7.2)	4.4 (3.1, 6.3)	<0.001	8.7 (6.2, 12.1)	4.9 (3.3, 6.6)	<0.001
Meeting RNI, n (%)	142 (83.5%)	40 (36.0%)	34 (29.3%)	47 (20.3%)	<0.001	182 (64.8%)	81 (23.3%)	<0.001
Meeting EAR, n (%)	168 (98.8%)	103 (92.8%)	99 (85.3%)	181 (78.0%)	<0.001	271 (96.4%)	280 (80.5%)	<0.001
Zinc, mg	7.57 (5.61, 10.21)	4.23 (3.30, 5.66)	3.57 (2.63, 4.92)	3.41 (2.51, 4.45)	<0.001	6.12 (4.23, 8.57)	3.49 (2.55, 4.57)	<0.001
Meeting RNI, n (%)	165 (97.1%)	95 (85.6%)	75 (64.7%)	138 (59.5%)	<0.001	260 (92.5%)	213 (61.2%)	<0.001
Meeting EAR, n (%)	168 (98.8%)	102 (91.9%)	92 (79.3%)	175 (75.4%)	<0.001	270 (96.1%)	267 (76.7%)	<0.001
Choline, mg	209.9 (119.0, 288.9)	160.2 (82.1, 229.0)	177.5 (84.4, 243.2)	152.1 (65.1, 237.9)	<0.001	191.3 (107.3, 273.0)	160.6 (68.3, 240.1)	<0.001
Meeting RNI, n (%)	92 (54.1%)	40 (36.0%)	48 (41.4%)	73 (31.5%)	<0.001	132 (47.0%)	121 (34.8%)	0.002
DHA, mg	55.2 (32.5, 87.1)	29.6 (15.7, 90.5)	28.1 (13.0, 60.1)	26.6 (9.6, 60.7)	<0.001	47 (24.1, 87.1)	26.9 (9.8, 60.3)	<0.001
**4-6 years**								
**Indonesia, n**	91	54	96	281		145	377	
Energy, kcal	1,482.3 (1,176.7, 1,817.4)	1,120 (938.3, 1,446.6)	1,096.5 (852.5, 1,384.7)	1,010.2 (765.4, 1,286.9)	<0.001	1,336.1 (1,087.4, 1,717.2)	1,040.4 (787.0, 1,330.6)	<0.001
Meeting EER, n (%)	52 (57.1%)	17 (31.5%)	21 (21.9%)	52 (18.5%)	<0.001	69 (47.6%)	73 (19.4%)	<0.001
Protein, g	47.1 (38.6, 59.9)	38.4 (31.2, 46.5)	33.3 (26.3, 42.1)	29.1 (20.7, 39.5)	<0.001	43.8 (35.6, 55.8)	30.6 (21.9, 40.0)	<0.001
Meeting RNI, n (%)	89 (97.8%)	47 (87.0%)	77 (80.2%)	178 (63.3%)	<0.001	136 (93.8%)	255 (67.6%)	<0.001
Carbohydrates, g	186.4 (152.8, 226.0)	139.4 (106.9, 177.8)	137.3 (105.6, 182.2)	124.7 (97.0, 166.8)	<0.001	170.7 (130.0, 214.7)	126.8 (99.6, 169.4)	<0.001
Meeting RNI, n (%)	26 (28.6%)	7 (13.0%)	12 (12.5%)	16 (5.7%)	<0.001	33 (22.8%)	28 (7.4%)	<0.001
Fat, g	56.7 (45.2, 76.5)	48.4 (36.6, 60.0)	43.9 (34.1, 58.5)	38.2 (27.1, 53.6)	<0.001	52.4 (43.5, 71.2)	40.3 (29.2, 56.9)	<0.001
Fiber, g	4.83 (2.90, 6.98)	4.68 (2.11, 6.52)	4.30 (2.46, 6.73)	3.34 (1.99, 5.76)	0.002	4.80 (2.64, 6.81)	3.52 (2.05, 6.03)	0.003
Meeting RNI, n (%)	0 (0.0%)	1 (1.9%)	0 (0.0%)	2 (0.7%)	0.43	1 (0.7%)	2 (0.5%)	>0.99

Vitamin A, μg RE	747.3 (566.3, 996.4)	478.1 (338.7, 677.3)	358.7 (210.9, 589.0)	277 (134.1, 619.6)	<0.001	639.4 (440.2, 940.2)	305.3 (147.0, 619.4)	<0.001
Meeting RNI, n (%)	79 (86.8%)	28 (51.9%)	38 (39.6%)	99 (35.2%)	<0.001	107 (73.8%)	137 (36.3%)	<0.001
Meeting EAR, n (%)	88 (96.7%)	41 (75.9%)	52 (54.2%)	132 (47.0%)	<0.001	129 (89.0%)	184 (48.8%)	<0.001
Vitamin B_1, mg	1.23 (0.93, 1.72)	0.76 (0.64, 0.99)	0.62 (0.44, 0.83)	0.40 (0.27, 0.81)	<0.001	1.00 (0.75, 1.47)	0.47 (0.30, 0.81)	<0.001
Meeting RNI, n (%)	87 (95.6%)	42 (77.8%)	52 (54.2%)	101 (35.9%)	<0.001	129 (89.0%)	153 (40.6%)	<0.001
Meeting EAR, n (%)	90 (98.9%)	49 (90.7%)	68 (70.8%)	118 (42.0%)	<0.001	139 (95.9%)	186 (49.3%)	<0.001
Vitamin B_2, mg	1.77 (1.38, 2.23)	1.20 (0.93, 1.44)	0.88 (0.68, 1.26)	0.60 (0.40, 0.88)	<0.001	1.53 (1.21, 2.05)	0.69 (0.45, 0.99)	<0.001
Meeting RNI, n (%)	91 (100.0%)	50 (92.6%)	77 (80.2%)	142 (50.5%)	<0.001	141 (97.2%)	219 (58.1%)	<0.001
Meeting EAR, n (%)	91 (100.0%)	51 (94.4%)	92 (95.8%)	182 (64.8%)	<0.001	142 (97.9%)	274 (72.7%)	<0.001
Vitamin B_{12}, μg	4.00 (2.85, 5.12)	2.98 (2.27, 4.24)	2.27 (1.72, 3.59)	1.86 (0.99, 3.35)	<0.001	3.63 (2.55, 4.81)	2.06 (1.14, 3.40)	<0.001
Meeting RNI, n (%)	90 (98.9%)	46 (85.2%)	79 (82.3%)	170 (60.5%)	<0.001	136 (93.8%)	249 (66.0%)	<0.001
Meeting EAR, n (%)	91 (100.0%)	48 (88.9%)	85 (88.5%)	180 (64.1%)	<0.001	139 (95.9%)	265 (70.3%)	<0.001
Vitamin C, mg	59.7 (43.0, 88.3)	20.7 (10.5, 50.4)	12 (4.7, 24.0)	7.7 (1.5, 25.2)	<0.001	50.2 (27.1, 76.2)	8.8 (2.1, 24.5)	<0.001
Meeting RNI, n (%)	66 (72.5%)	15 (27.8%)	10 (10.4%)	38 (13.5%)	<0.001	81 (55.9%)	48 (12.7%)	<0.001
Meeting EAR, n (%)	73 (80.2%)	17 (31.5%)	13 (13.5%)	47 (16.7%)	<0.001	90 (62.1%)	60 (15.9%)	<0.001
Vitamin D, μg	10.7 (8.2, 14.8)	5.3 (3.6, 9.0)	4.6 (3.5, 5.9)	1.3 (0.6, 2.5)	<0.001	9.2 (5.3, 13.6)	2 (0.7, 4.1)	<0.001
Meeting RNI, n (%)	22 (24.2%)	2 (3.7%)	1 (1.0%)	2 (0.7%)	<0.001	24 (16.6%)	3 (0.8%)	<0.001
Meeting EAR, n (%)	49 (53.8%)	10 (18.5%)	6 (6.3%)	5 (1.8%)	<0.001	59 (40.7%)	11 (2.9%)	<0.001
Calcium, mg	1,084.4 (841.6, 1,393.1)	674.9 (485.1, 972.5)	511.1 (405.5, 635.1)	232.8 (156.3, 336.1)	<0.001	952.7 (667.6, 1,251.6)	285.6 (181.6, 444.6)	<0.001
Meeting RNI, n (%)	56 (61.5%)	11 (20.4%)	2 (2.1%)	0 (0.0%)	<0.001	67 (46.2%)	2 (0.5%)	<0.001
Meeting EAR, n (%)	70 (76.9%)	19 (35.2%)	8 (8.3%)	1 (0.4%)	<0.001	89 (61.4%)	9 (2.4%)	<0.001
Iron, mg	13.7 (10.3, 18.0)	7.5 (5.8, 11.4)	6.8 (4.7, 8.5)	5.6 (4.0, 7.8)	<0.001	11.8 (7.6, 15.8)	5.9 (4.1, 8.0)	<0.001
Meeting RNI, n (%)	70 (76.9%)	18 (33.3%)	15 (15.6%)	28 (10.0%)	<0.001	88 (60.7%)	43 (11.4%)	<0.001
Meeting EAR, n (%)	87 (95.6%)	49 (90.7%)	78 (81.3%)	204 (72.6%)	<0.001	136 (93.8%)	282 (74.8%)	<0.001
Zinc, mg	8.85 (6.44, 11.41)	4.84 (3.46, 7.08)	4.05 (3.35, 5.50)	3.97 (2.83, 5.22)	<0.001	7.08 (5.02, 10.07)	4.00 (2.93, 5.32)	<0.001
Meeting RNI, n (%)	83 (91.2%)	26 (48.1%)	32 (33.3%)	75 (26.7%)	<0.001	109 (75.2%)	107 (28.4%)	<0.001
Meeting EAR, n (%)	87 (95.6%)	35 (64.8%)	51 (53.1%)	139 (49.5%)	<0.001	122 (84.1%)	190 (50.4%)	<0.001
Choline, mg	279.9 (199.1, 356.1)	200.8 (114.1, 261.5)	219.7 (121.6, 360.1)	187.1 (88.7, 266.6)	<0.001	252.8 (156.5, 325.7)	192.6 (93.7, 287.5)	<0.001
Meeting RNI, n (%)	59 (64.8%)	17 (31.5%)	37 (38.5%)	81 (28.8%)	<0.001	76 (52.4%)	118 (31.3%)	<0.001
DHA, mg	52.6 (27.7, 85.7)	28.4 (8.0, 59.0)	31.2 (19.7, 55.8)	26.2 (11.8, 58.4)	<0.001	40.7 (22.3, 77.7)	27 (14.0, 57.8)	<0.001
**7-12- years**								

Indonesia, n	25	77	125	838		102	963	
Energy, kcal	1,535.1 (1,191.9, 1,924.7)	1,501 (1,231.4, 1,799.5)	1,352.2 (1,078.9, 1,667.0)	1,213.9 (941.9, 1,525.0)	<0.001	1,506.4 (1,219.2, 1,799.8)	1,227.1 (953.9, 1,545.9)	<0.001
Meeting EER, n (%)	9 (36.0%)	23 (29.9%)	26 (20.8%)	114 (13.6%)	<0.001	32 (31.4%)	140 (14.5%)	<0.001
Protein, g	49.7 (41.1, 63.0)	47.4 (37.9, 58.2)	40.8 (32.3, 50.7)	36.5 (27.4, 48.2)	<0.001	47.8 (39.1, 59.3)	37.1 (28.3, 48.8)	<0.001
Meeting RNI, n (%)	18 (72.0%)	42 (54.5%)	49 (39.2%)	261 (31.1%)	<0.001	60 (58.8%)	310 (32.2%)	<0.001
Carbohydrates, g	180 (139.1, 240.9)	190.2 (151.1, 234.9)	171.8 (125.8, 212.9)	150.9 (115.4, 195.3)	<0.001	189.6 (150.5, 236.4)	153.7 (116.9, 197.9)	<0.001
Meeting RNI, n (%)	3 (12.0%)	11 (14.3%)	11 (8.8%)	69 (8.2%)	0.27	14 (13.7%)	80 (8.3%)	0.067
Fat, g	63.5 (51.8, 75.8)	58.3 (45.0, 74.7)	53.2 (39.3, 74.3)	47.1 (34.7, 63.4)	<0.001	59.9 (46.2, 75.8)	47.8 (34.9, 64.3)	<0.001
Fiber, g	4.89 (2.23, 8.75)	4.90 (3.21, 8.06)	5.52 (3.19, 8.15)	4.48 (2.70, 7.17)	0.035	4.89 (2.92, 8.45)	4.56 (2.74, 7.27)	0.10
Meeting RNI, n (%)	0 (0.0%)	0 (0.0%)	0 (0.0%)	1 (0.1%)	>0.99	0 (0.0%)	1 (0.1%)	>0.99
Vitamin A, µg RE	596.8 (428.9, 791.7)	418.1 (320.6, 648.7)	377.5 (255.3, 588.0)	251.8 (120.0, 470.3)	<0.001	486.6 (339.6, 714.0)	277.7 (138.6, 482.7)	<0.001
Meeting RNI, n (%)	14 (56.0%)	31 (40.3%)	34 (27.2%)	166 (19.8%)	<0.001	45 (44.1%)	200 (20.8%)	<0.001
Meeting EAR, n (%)	20 (80.0%)	43 (55.8%)	57 (45.6%)	268 (32.0%)	<0.001	63 (61.8%)	325 (33.7%)	<0.001
Vitamin B_1, mg	1.35 (1.06, 1.87)	0.91 (0.70, 1.17)	0.64 (0.45, 0.87)	0.43 (0.28, 0.67)	<0.001	0.99 (0.75, 1.34)	0.45 (0.30, 0.70)	<0.001
Meeting RNI, n (%)	23 (92.0%)	35 (45.5%)	18 (14.4%)	79 (9.4%)	<0.001	58 (56.9%)	97 (10.1%)	<0.001
Meeting EAR, n (%)	25 (100.0%)	47 (61.0%)	44 (35.2%)	147 (17.5%)	<0.001	72 (70.6%)	191 (19.8%)	<0.001
Vitamin B_2, mg	1.60 (1.33, 1.98)	1.18 (0.91, 1.52)	0.91 (0.71, 1.25)	0.62 (0.43, 0.89)	<0.001	1.30 (0.98, 1.65)	0.66 (0.45, 0.96)	<0.001
Meeting RNI, n (%)	23 (92.0%)	47 (61.0%)	56 (44.8%)	160 (19.1%)	<0.001	70 (68.6%)	216 (22.4%)	<0.001
Meeting EAR, n (%)	24 (96.0%)	58 (75.3%)	82 (65.6%)	260 (31.0%)	<0.001	82 (80.4%)	342 (35.5%)	<0.001
Vitamin B_{12}, µg	3.25 (2.60, 4.71)	2.82 (2.12, 4.00)	2.77 (1.58, 4.45)	1.95 (1.18, 3.22)	<0.001	2.99 (2.19, 4.08)	2.06 (1.22, 3.38)	<0.001
Meeting RNI, n (%)	18 (72.0%)	44 (57.1%)	75 (60.0%)	306 (36.5%)	<0.001	62 (60.8%)	381 (39.6%)	<0.001
Meeting EAR, n (%)	20 (80.0%)	56 (72.7%)	84 (67.2%)	376 (44.9%)	<0.001	76 (74.5%)	460 (47.8%)	<0.001
Vitamin C, mg	83 (26.2, 121.7)	33.4 (17.0, 53.6)	15.1 (5.8, 35.5)	8.9 (2.2, 22.6)	<0.001	35.4 (19.1, 66.9)	9.3 (2.7, 23.5)	<0.001
Meeting RNI, n (%)	15 (60.0%)	26 (33.8%)	24 (19.2%)	89 (10.6%)	<0.001	41 (40.2%)	113 (11.7%)	<0.001
Meeting EAR, n (%)	15 (60.0%)	32 (41.6%)	29 (23.2%)	100 (11.9%)	<0.001	47 (46.1%)	129 (13.4%)	<0.001
Vitamin D, µg	11.4 (8.7, 13.8)	5.5 (4.7, 7.7)	4.3 (3.2, 5.4)	1.3 (0.6, 2.2)	<0.001	6 (4.8, 9.6)	1.4 (0.7, 2.8)	<0.001
Meeting RNI, n (%)	6 (24.0%)	1 (1.3%)	0 (0.0%)	9 (1.1%)	<0.001	7 (6.9%)	9 (0.9%)	<0.001
Meeting EAR, n (%)	16 (64.0%)	6 (7.8%)	2 (1.6%)	14 (1.7%)	<0.001	22 (21.6%)	16 (1.7%)	<0.001
Calcium, mg	991.8 (895.1, 1,334.5)	644.5 (474.2, 827.4)	505.2 (396.7, 687.4)	259.4 (180.2, 400.0)	<0.001	777.1 (484.7, 966.5)	279.9 (191.5, 438.5)	<0.001
Meeting RNI, n (%)	12 (48.0%)	6 (7.8%)	1 (0.8%)	9 (1.1%)	<0.001	18 (17.6%)	10 (1.0%)	<0.001

Meeting EAR, n (%)	16 (64.0%)	17 (22.1%)	9 (7.2%)	12 (1.4%)	<0.001	33 (32.4%)	21 (2.2%)	<0.001
Iron, mg	13.4 (11.7, 16.4)	9 (7.7, 12.0)	7.2 (5.0, 9.5)	6.7 (4.8, 9.1)	<0.001	10 (7.9, 13.5)	6.8 (4.8, 9.1)	<0.001
Meeting RNI, n (%)	22 (88.0%)	59 (76.6%)	67 (53.6%)	402 (48.0%)	<0.001	81 (79.4%)	469 (48.7%)	<0.001
Meeting EAR, n (%)	23 (92.0%)	67 (87.0%)	90 (72.0%)	591 (70.5%)	0.002	90 (88.2%)	681 (70.7%)	<0.001
Zinc, mg	8 (7.2, 9.6)	6.6 (5.1, 7.8)	5 (3.8, 6.5)	4.7 (3.4, 6.3)	<0.001	6.90 (5.42, 8.70)	4.72 (3.48, 6.38)	<0.001
Meeting RNI, n (%)	18 (72.0%)	40 (51.9%)	39 (31.2%)	225 (26.8%)	<0.001	58 (56.9%)	264 (27.4%)	<0.001
Meeting EAR, n (%)	22 (88.0%)	44 (57.1%)	41 (32.8%)	266 (31.7%)	<0.001	66 (64.7%)	307 (31.9%)	<0.001
Choline, mg	277.9 (177.9, 364.7)	241.5 (144.2, 376.1)	219.7 (119.5, 315.6)	199.3 (110.9, 290.0)	<0.001	242.5 (155.4, 376.1)	201.1 (112.9, 293.4)	<0.001
Meeting RNI, n (%)	6 (24.0%)	20 (26.0%)	23 (18.4%)	93 (11.1%)	<0.001	26 (25.5%)	116 (12.0%)	<0.001
DHA, mg	42.5 (19.7, 134.1)	39.3 (21.7, 72.4)	28.2 (16.3, 59.0)	29.8 (13.4, 65.5)	0.24	39.4 (20.4, 82.9)	29.7 (13.6, 64.1)	0.044

	High dairy consumer	Middle dairy consumer	Low dairy consumer	No dairy consumer	*p*-Value	Meeting daily dairy recommendations	Not meeting daily dairy recommendations	*p*-Value
**2-3 years**								
**Malaysia, n**	179	75	32	41		180	**147**	
Energy, kcal	1,258.8 (1,075.1, 1,516.6)	1,099.7 (929.3, 1,299.7)	1,157.3 (998.3, 1,597.0)	1,195.3 (969.6, 1,387.5)	<0.001	1,260.2 (1,080.4, 1,517.3)	1,144 (942.7, 1,345.7)	<0.001
Meeting EER, n (%)	161 (89.9%)	55 (73.3%)	26 (81.3%)	32 (78.0%)	0.007	162 (90.0%)	112 (76.2%)	<0.001
Protein, g	46.5 (38.7, 57.7)	38.2 (32.6, 50.9)	42.9 (32.1, 53.8)	42.5 (33.8, 49.7)	0.004	46.5 (38.7, 57.8)	40.3 (32.6, 51.6)	<0.001
Meeting RNI, n (%)	179 (100.0%)	75 (100.0%)	32 (100.0%)	41 (100.0%)	1	180 (100.0%)	147 (100.0%)	1
Carbohydrates, g	167.5 (145.0, 208.8)	146.2 (122.1, 171.6)	161.8 (130.6, 213.8)	162.3 (115.4, 207.8)	0.002	168.3 (145.3, 209.1)	153.8 (121.2, 187.6)	<0.001
Fat, g	44.2 (34.8, 51.7)	36.5 (28.7, 46.1)	42.1 (30.8, 55.3)	42.7 (29.9, 57.9)	0.009	44.3 (34.9, 52.0)	38.1 (29.7, 48.5)	0.007
Meeting RNI, n (%)	163 (91.1%)	66 (88.0%)	29 (90.6%)	38 (92.7%)	0.85	164 (91.1%)	132 (89.8%)	0.69
Vitamin A, μg RE	759.4 (597.0, 946.7)	623.2 (416.9, 847.8)	568 (439.4, 750.9)	363.8 (227.0, 556.3)	<0.001	761 (600.9, 949.3)	548.4 (336.3, 724.0)	<0.001
Meeting RNI, n (%)	170 (95.0%)	58 (77.3%)	25 (78.1%)	16 (39.0%)	<0.001	171 (95.0%)	98 (66.7%)	<0.001
Meeting EAR, n (%)	177 (98.9%)	69 (92.0%)	32 (100.0%)	27 (65.9%)	<0.001	178 (98.9%)	127 (86.4%)	<0.001
β-carotene, μg	769.4 (420.2, 1,415.1)	664.1 (325.5, 1,664.9)	991.7 (264.4, 1,653.1)	253.5 (83.2, 671.0)	<0.001	767.4 (414.7, 1,407.1)	634.2 (169.5, 1,578.8)	0.010
Vitamin B_1, mg	1.11 (0.88, 1.47)	0.92 (0.59, 1.25)	0.88 (0.59, 1.05)	0.64 (0.43, 0.82)	<0.001	1.12 (0.88, 1.47)	0.77 (0.54, 1.07)	<0.001
Meeting RNI, n (%)	177 (98.9%)	66 (88.0%)	29 (90.6%)	27 (65.9%)	<0.001	178 (98.9%)	121 (82.3%)	<0.001
Meeting EAR, n (%)	177 (98.9%)	71 (94.7%)	30 (93.8%)	34 (82.9%)	<0.001	178 (98.9%)	134 (91.2%)	<0.001
Vitamin B_2, mg	1.55 (1.21, 1.97)	1.22 (0.94, 1.51)	1.15 (0.87, 1.40)	0.85 (0.63, 1.24)	<0.001	1.55 (1.21, 1.99)	1.10 (0.83, 1.47)	<0.001
Meeting RNI, n (%)	179 (100.0%)	75 (100.0%)	32 (100.0%)	36 (87.8%)	<0.001	180 (100.0%)	142 (96.6%)	0.018
Meeting EAR, n (%)	179 (100.0%)	75 (100.0%)	32 (100.0%)	39 (95.1%)	0.025	180 (100.0%)	145 (98.6%)	0.20
Vitamin B_3, mg	11.3 (8.9, 14.3)	9.1 (6.8, 11.3)	8.5 (5.5, 11.1)	7.7 (5.9, 12.3)	<0.001	11.3 (8.9, 14.4)	8.9 (6.0, 11.2)	<0.001
Vitamin B_{12}, μg	2.92 (2.07, 4.36)	1.84 (1.25, 2.74)	1.53 (0.96, 3.42)	1.43 (0.62, 2.14)	<0.001	2.92 (2.07, 4.31)	1.69 (1.02, 2.77)	<0.001
Meeting RNI, n (%)	166 (92.7%)	49 (65.3%)	16 (50.0%)	20 (48.8%)	<0.001	167 (92.8%)	84 (57.1%)	<0.001
Vitamin C, mg	114.6 (77.9, 161.7)	58.2 (45.1, 85.5)	34.9 (27.2, 84.7)	26.3 (16.4, 68.7)	<0.001	114.5 (77.2, 161.3)	50.7 (29.0, 79.2)	<0.001
Meeting RNI, n (%)	175 (97.8%)	66 (88.0%)	21 (65.6%)	19 (46.3%)	<0.001	176 (97.8%)	105 (71.4%)	<0.001
Meeting EAR, n (%)	177 (98.9%)	70 (93.3%)	27 (84.4%)	22 (53.7%)	<0.001	178 (98.9%)	118 (80.3%)	<0.001
Vitamin D, μg	9.8 (7.2, 13.4)	5.3 (3.7, 7.1)	4.5 (2.9, 6.2)	1.6 (0.6, 3.2)	<0.001	9.8 (7.1, 13.4)	4.3 (2.6, 6.3)	<0.001
Meeting RNI, n (%)	28 (15.6%)	0 (0.0%)	0 (0.0%)	0 (0.0%)	<0.001	28 (15.6%)	0 (0.0%)	<0.001

Meeting EAR, n (%)	86 (48.0%)	8 (10.7%)	1 (3.1%)	1 (2.4%)	<0.001	86 (47.8%)	10 (6.8%)	<0.001
Calcium, mg	936.5 (776.6, 1,184.1)	584.8 (496.9, 718.1)	472.7 (369.0, 563.3)	278.9 (206.0, 498.2)	<0.001	937 (778.9, 1,184.3)	506.1 (382.3, 638.8)	<0.001
Meeting RNI, n (%)	147 (82.1%)	19 (25.3%)	5 (15.6%)	3 (7.3%)	<0.001	148 (82.2%)	26 (17.7%)	<0.001
Meeting EAR, n (%)	177 (98.9%)	55 (73.3%)	13 (40.6%)	10 (24.4%)	<0.001	178 (98.9%)	77 (52.4%)	<0.001
Iron, mg	13.1 (10.5, 16.5)	10 (7.7, 12.3)	8.4 (6.9, 11.4)	7.1 (5.4, 11.6)	<0.001	13.1 (10.5, 16.5)	9 (6.8, 11.8)	<0.001
Meeting RNI, n (%)	179 (100.0%)	75 (100.0%)	32 (100.0%)	38 (92.7%)	0.003	180 (100.0%)	144 (98.0%)	0.090
Meeting EAR, n (%)	179 (100.0%)	75 (100.0%)	32 (100.0%)	41 (100.0%)	1	180 (100.0%)	147 (100.0%)	1
Sodium, mg	1,104.9 (889.7, 1,695.3)	1,231.7 (937.2, 1,738.3)	1,318.2 (994.2, 1,982.8)	1,353.2 (1,032.9, 1,625.5)	0.35	1,106.3 (892.3, 1,693.9)	1,281.7 (980.3, 1,738.3)	0.17
Meeting RNI, n (%)	114 (63.7%)	53 (70.7%)	24 (75.0%)	31 (75.6%)	0.31	115 (63.9%)	107 (72.8%)	0.086
Potassium, mg	1,331.1 (1,065.6, 1,640.1)	959.7 (791.0, 1,156.1)	844.4 (622.9, 1,151.7)	761.6 (524.1, 985.3)	<0.001	1,338.9 (1,077.5, 1,628.2)	880.7 (718.6, 1,126.8)	<0.001
Meeting RNI, n (%)	1 (0.6%)	0 (0.0%)	0 (0.0%)	0 (0.0%)	>0.99	1 (0.6%)	0 (0.0%)	>0.99
Phosphorus, mg	824.7 (682.7, 1,017.3)	591.9 (486.6, 729.9)	551.3 (448.4, 764.1)	466.9 (340.1, 602.1)	<0.001	828.5 (684.8, 1,027.7)	563.2 (443.0, 684.2)	<0.001
Meeting RNI, n (%)	176 (98.3%)	63 (84.0%)	24 (75.0%)	21 (51.2%)	<0.001	177 (98.3%)	107 (72.8%)	<0.001
**4-6 years**								
**Malaysia, n**	189	155	170	255		190	579	
Energy, kcal	1,520.6 (1,246.3, 1,813.2)	1,298.1 (1,103.5, 1,618.3)	1,231.6 (1,037.8, 1,507.9)	1,188.9 (922.2, 1,486.9)	<0.001	1,516.9 (1,246.3, 1,813.2)	1,238.1 (997.7, 1,527.4)	<0.001
Meeting EER, n (%)	145 (76.7%)	86 (55.5%)	83 (48.8%)	113 (44.3%)	<0.001	146 (76.8%)	281 (48.5%)	<0.001
Protein, g	53.9 (43.4, 65.5)	50.1 (39.9, 61.8)	44.3 (35.7, 59.6)	42.7 (31.5, 54.8)	<0.001	53.8 (42.4, 65.5)	44.7 (34.4, 58.7)	<0.001
Meeting RNI, n (%)	189 (100.0%)	154 (99.4%)	169 (99.4%)	251 (98.4%)	0.33	190 (100.0%)	573 (99.0%)	0.35
Carbohydrates, g	200.9 (172.2, 245.9)	170.6 (145.2, 210.4)	164.2 (135.6, 210.8)	158.3 (128.6, 202.3)	<0.001	199.7 (171.0, 245.9)	162.2 (132.9, 206.7)	<0.001
Fat, g	51.6 (40.9, 63.8)	46.4 (33.5, 58.3)	42.4 (31.6, 57.8)	39 (28.4, 52.6)	<0.001	51.7 (40.9, 63.8)	41.5 (31.2, 55.7)	<0.001
Meeting RNI, n (%)	159 (84.1%)	115 (74.2%)	114 (67.1%)	153 (60.0%)	<0.001	160 (84.2%)	381 (65.8%)	<0.001
Vitamin A, μg RE	840.3 (596.4, 1,065.9)	599 (475.8, 857.4)	532.6 (411.3, 721.4)	491.9 (292.4, 700.3)	<0.001	838.9 (596.4, 1,065.9)	531.2 (381.5, 742.9)	<0.001
Meeting RNI, n (%)	174 (92.1%)	123 (79.4%)	117 (68.8%)	142 (55.7%)	<0.001	175 (92.1%)	381 (65.8%)	<0.001
Meeting EAR, n (%)	187 (98.9%)	141 (91.0%)	152 (89.4%)	183 (71.8%)	<0.001	188 (98.9%)	475 (82.0%)	<0.001
β-carotene, μg	676.9 (302.9, 1,381.3)	512 (197.4, 1,281.5)	506.3 (161.3, 1,212.9)	381.4 (109.3, 1,123.6)	<0.001	667.8 (301.6, 1,381.3)	474.8 (150.3, 1,199.2)	<0.001

Vitamin B_1, mg	1.33 (0.92, 1.80)	1.08 (0.80, 1.45)	0.94 (0.66, 1.28)	0.72 (0.48, 1.00)	<0.001	1.32 (0.91, 1.80)	0.88 (0.60, 1.26)	<0.001
Meeting RNI, n (%)	179 (94.7%)	140 (90.3%)	136 (80.0%)	158 (62.0%)	<0.001	180 (94.7%)	433 (74.8%)	<0.001
Meeting EAR, n (%)	188 (99.5%)	146 (94.2%)	146 (85.9%)	186 (72.9%)	<0.001	189 (99.5%)	477 (82.4%)	<0.001
Vitamin B_2, mg	1.88 (1.33, 2.32)	1.42 (1.13, 1.79)	1.20 (0.94, 1.62)	0.98 (0.68, 1.42)	<0.001	1.87 (1.33, 2.32)	1.19 (0.84, 1.64)	<0.001
Meeting RNI, n (%)	189 (100.0%)	151 (97.4%)	155 (91.2%)	216 (84.7%)	<0.001	190 (100.0%)	521 (90.0%)	<0.001
Meeting EAR, n (%)	189 (100.0%)	154 (99.4%)	167 (98.2%)	229 (89.8%)	<0.001	190 (100.0%)	549 (94.8%)	0.001
Vitamin B_3, mg	13.3 (10.3, 18.0)	11.6 (8.5, 15.0)	10.9 (7.6, 13.7)	8.6 (5.9, 12.1)	<0.001	13.2 (10.3, 18.0)	9.9 (7.1, 13.5)	<0.001
Vitamin B_{12}, μg	3.13 (2.28, 4.59)	2.49 (1.49, 3.92)	2.03 (1.12, 3.69)	1.90 (1.07, 3.42)	<0.001	3.13 (2.27, 4.59)	2.08 (1.18, 3.54)	<0.001
Meeting RNI, n (%)	171 (90.5%)	116 (74.8%)	112 (65.9%)	157 (61.6%)	<0.001	171 (90.0%)	385 (66.5%)	<0.001
Vitamin C, mg	102.9 (63.5, 158.8)	56.5 (34.2, 95.4)	41.7 (25.2, 73.7)	31.2 (14.9, 56.2)	<0.001	102.8 (63.0, 158.8)	40.5 (22.2, 70.0)	<0.001
Meeting RNI, n (%)	182 (96.3%)	131 (84.5%)	116 (68.2%)	128 (50.2%)	<0.001	182 (95.8%)	375 (64.8%)	<0.001
Meeting EAR, n (%)	186 (98.4%)	134 (86.5%)	128 (75.3%)	149 (58.4%)	<0.001	186 (97.9%)	411 (71.0%)	<0.001
Vitamin D, μg	10.8 (7.8, 14.8)	6.1 (4.1, 7.8)	4 (2.5, 5.7)	2.4 (0.9, 4.0)	<0.001	10.7 (7.8, 14.8)	3.7 (2.0, 6.1)	<0.001
Meeting RNI, n (%)	42 (22.2%)	1 (0.6%)	4 (2.4%)	2 (0.8%)	<0.001	42 (22.1%)	7 (1.2%)	<0.001
Meeting EAR, n (%)	112 (59.3%)	15 (9.7%)	14 (8.2%)	11 (4.3%)	<0.001	112 (58.9%)	40 (6.9%)	<0.001
Calcium, mg	1,042.3 (791.7, 1,398.7)	674.2 (542.1, 813.6)	508.1 (403.5, 630.8)	348.5 (215.4, 461.8)	<0.001	1,037.1 (791.7, 1,398.7)	474.1 (331.1, 650.5)	<0.001
Meeting RNI, n (%)	97 (51.3%)	15 (9.7%)	5 (2.9%)	1 (0.4%)	<0.001	97 (51.1%)	21 (3.6%)	<0.001
Meeting EAR, n (%)	141 (74.6%)	41 (26.5%)	11 (6.5%)	7 (2.7%)	<0.001	142 (74.7%)	58 (10.0%)	<0.001
Iron, mg	15.3 (11.9, 18.4)	10.9 (9.2, 13.4)	10.1 (7.8, 13.5)	8.7 (6.4, 12.2)	<0.001	15.2 (11.9, 18.4)	9.9 (7.5, 13.0)	<0.001
Meeting RNI, n (%)*	189 (100.0%)	154 (99.4%)	166 (97.6%)	245 (96.1%)	0.009	190 (100.0%)	564 (97.4%)	0.029
Meeting EAR, n (%)*	189 (100.0%)	155 (100.0%)	169 (99.4%)	251 (98.4%)	0.16	190 (100.0%)	574 (99.1%)	0.34
Sodium, mg	1,550.7 (1,146.1, 1,942.4)	1,505.1 (1,097.3, 2,233.9)	1,670.2 (1,212.8, 2,189.2)	1,504.1 (1,092.9, 2,154.1)	0.40	1,544.5 (1,145.6, 1,942.4)	1,531.2 (1,130.4, 2,180.4)	0.69
Meeting RNI, n (%)	135 (71.4%)	109 (70.3%)	130 (76.5%)	173 (67.8%)	0.29	135 (71.1%)	412 (71.2%)	0.98
Potassium, mg	1,488.1 (1,133.2, 1,838.1)	1,082.9 (853.6, 1,363.4)	866.7 (708.8, 1,117.7)	794.6 (550.8, 1,072.2)	<0.001	1,486.9 (1,127.0, 1,838.1)	891.7 (674.5, 1,174.5)	<0.001
Meeting RNI, n (%)	0 (0.0%)	0 (0.0%)	0 (0.0%)	0 (0.0%)	1	0 (0%)	0 (0%)	1
Phosphorus, mg	922.7 (756.7, 1,132.1)	732.9 (580.9, 885.5)	597.1 (470.1, 760.0)	496.2 (369.4, 685.9)	<0.001	931.6 (756.7, 1,132.1)	595.5 (430.4, 798.3)	<0.001
Meeting RNI, n (%)	181 (95.8%)	132 (85.2%)	115 (67.6%)	127 (49.8%)	<0.001	182 (95.8%)	373 (64.4%)	<0.001
**7-12 years**								
**Malaysia, n**	49	193	336	774		53	1299	

Energy, kcal	1,699.3 (1,429.4, 2,019.1)	1,605.6 (1,355.9, 1,944.0)	1,494.5 (1,194.9, 1,777.8)	1,407.7 (1,125.1, 1,761.2)	<0.001	1,714.6 (1,429.4, 2,019.1)	1,458.1 (1,175.5, 1,793.2)	<0.001
Meeting EER, n (%)	22 (44.9%)	68 (35.2%)	94 (28.0%)	184 (23.8%)	<0.001	25 (47.2%)	343 (26.4%)	<0.001
Protein, g	57.1 (49.3, 74.0)	60.9 (49.5, 76.3)	53.4 (40.8, 67.7)	50.9 (38.2, 66.3)	<0.001	57.1 (49.6, 74.0)	53.3 (40.2, 68.3)	0.016
Meeting RNI, n (%)	49 (100.0%)	191 (99.0%)	316 (94.0%)	714 (92.2%)	<0.001	53 (100.0%)	1,217 (93.7%)	0.071
Carbohydrates, g	229.7 (192.8, 271.9)	216.2 (176.9, 260.4)	199.7 (159.6, 252.2)	192.1 (150.5, 242.1)	<0.001	229.6 (192.8, 271.9)	197.6 (157.1, 248.1)	0.003
Fat, g	58.9 (42.5, 70.0)	57.3 (42.3, 72.1)	51 (38.0, 63.5)	45.8 (34.1, 61.5)	<0.001	60.2 (42.5, 74.2)	48.7 (35.7, 63.7)	<0.001
Meeting RNI, n (%)	35 (71.4%)	129 (66.8%)	181 (53.9%)	345 (44.6%)	<0.001	38 (71.7%)	652 (50.2%)	0.002
Vitamin A, μg RE	1,080.5 (765.0, 1,353.8)	868.8 (654.2, 1,184.7)	744.4 (462.3, 1,034.9)	525.3 (342.9, 801.0)	<0.001	1,080.5 (764.7, 1,353.8)	628.1 (396.2, 944.9)	<0.001
Meeting RNI, n (%)	643.1 (272.2, 1,628.0)	1,298.9 (406.7, 2,728.0)	1,011.4 (319.2, 2,416.1)	664.7 (258.5, 1,616.0)	<0.001	50 (94.3%)	747 (57.5%)	<0.001
Meeting EAR, n (%)	46 (93.9%)	159 (82.4%)	228 (67.9%)	364 (47.0%)	<0.001	52 (98.1%)	979 (75.4%)	<0.001
β-carotene, μg	48 (98.0%)	182 (94.3%)	273 (81.3%)	528 (68.2%)	<0.001	642.6 (272.2, 1,628.0)	812.6 (297.0, 1,980.5)	0.49
Vitamin B_1, mg	1.33 (0.92, 1.80)	1.08 (0.80, 1.45)	0.94 (0.66, 1.28)	0.72 (0.48, 1.00)	<0.001	2.06 (1.52, 2.70)	1.00 (0.65, 1.44)	<0.001
Meeting RNI, n (%)	45 (91.8%)	158 (81.9%)	218 (64.9%)	262 (33.9%)	<0.001	49 (92.5%)	634 (48.8%)	<0.001
Meeting EAR, n (%)	45 (91.8%)	171 (88.6%)	256 (76.2%)	389 (50.3%)	<0.001	49 (92.5%)	812 (62.5%)	<0.001
Vitamin B_2, mg	1.88 (1.33, 2.32)	1.42 (1.13, 1.79)	1.20 (0.94, 1.62)	0.98 (0.68, 1.42)	<0.001	2.17 (1.79, 2.68)	1.25 (0.88, 1.73)	<0.001
Meeting RNI, n (%)	47 (95.9%)	177 (91.7%)	254 (75.6%)	413 (53.4%)	<0.001	51 (96.2%)	840 (64.7%)	<0.001
Meeting EAR, n (%)	48 (98.0%)	186 (96.4%)	302 (89.9%)	561 (72.5%)	<0.001	52 (98.1%)	1,045 (80.4%)	0.001
Vitamin B_3, mg	15.4 (11.6, 19.8)	13.7 (9.9, 18.4)	12.2 (8.8, 16.8)	10 (6.9, 13.9)	<0.001	15.4 (11.7, 19.7)	11.1 (7.6, 15.3)	<0.001
Vitamin B_{12}, μg	3.13 (2.28, 4.59)	2.49 (1.49, 3.92)	2.03 (1.12, 3.69)	1.90 (1.07, 3.42)	<0.001	3.59 (2.39, 5.09)	2.25 (1.18, 3.86)	<0.001
Meeting RNI, n (%)	31 (63.3%)	84 (43.5%)	126 (37.5%)	280 (36.2%)	<0.001	34 (64.2%)	487 (37.5%)	<0.001
Vitamin C, mg	103.9 (68.0, 152.6)	77.8 (46.2, 132.5)	60.7 (34.6, 107.3)	39.6 (21.0, 79.4)	<0.001	103.9 (67.1, 153.2)	48.4 (26.3, 94.7)	<0.001
Meeting RNI, n (%)	27 (55.1%)	92 (47.7%)	128 (38.1%)	197 (25.5%)	<0.001	29 (54.7%)	415 (31.9%)	<0.001
Meeting EAR, n (%)	44 (89.8%)	153 (79.3%)	237 (70.5%)	382 (49.4%)	<0.001	46 (86.8%)	770 (59.3%)	<0.001
Vitamin D, μg	9.9 (7.6, 15.8)	7.1 (4.0, 9.6)	4.4 (2.5, 6.6)	2.4 (1.2, 4.3)	<0.001	9.9 (7.6, 15.8)	3.3 (1.7, 5.9)	<0.001
Meeting RNI, n (%)	14 (28.6%)	8 (4.1%)	2 (0.6%)	3 (0.4%)	<0.001	14 (26.4%)	13 (1.0%)	<0.001
Meeting EAR, n (%)	23 (46.9%)	47 (24.4%)	29 (8.6%)	24 (3.1%)	<0.001	25 (47.2%)	98 (7.5%)	<0.001
Calcium, mg	1,134.8 (871.8, 1,408.6)	813.1 (637.2, 995.1)	598.2 (457.1, 803.4)	403.3 (268.9, 565.8)	<0.001	1,131.2 (871.8, 1,408.6)	504.8 (330.1, 711.1)	<0.001
Meeting RNI, n (%)	24 (49.0%)	27 (14.0%)	18 (5.4%)	14 (1.8%)	<0.001	25 (47.2%)	58 (4.5%)	<0.001

Meeting EAR, n (%)	41 (83.7%)	68 (35.2%)	57 (17.0%)	32 (4.1%)	<0.001	43 (81.1%)	155 (11.9%)	<0.001
Iron, mg	16.2 (13.1, 18.7)	12.9 (10.0, 17.5)	11.7 (8.6, 15.8)	9.8 (7.1, 13.7)	<0.001	16.2 (13.1, 19.7)	10.9 (7.8, 14.7)	<0.001
Meeting RNI, n (%)*	43 (87.8%)	152 (79.2%)	253 (75.7%)	420 (54.7%)	<0.001	46 (86.8%)	822 (63.7%)	<0.001
Meeting EAR, n (%)*	44 (89.8%)	164 (85.4%)	274 (82.0%)	517 (67.3%)	<0.001	48 (90.6%)	951 (73.7%)	0.006
Sodium, mg	1,937 (1,346.4, 2,669.5)	1,944.5 (1,478.1, 2,718.9)	1,804.3 (1,248.2, 2,508.9)	1,776.2 (1,188.0, 2,538.6)	0.010	1,989.2 (1,395.2, 2,797.7)	1,816.8 (1,239.5, 2,556.2)	0.13
Meeting RNI, n (%)	39 (79.6%)	153 (79.3%)	235 (69.9%)	497 (64.2%)	<0.001	43 (81.1%)	881 (67.8%)	0.041
Potassium, mg	1,532.2 (1,232.0, 1,990.6)	1,262.5 (1,025.4, 1,659.2)	1,095 (834.5, 1,405.1)	885.6 (652.9, 1,216.1)	<0.001	1,507.1 (1,232.0, 1,990.6)	1,004.5 (731.3, 1,332.9)	<0.001
Meeting RNI, n (%)	0 (0.0%)	1 (0.5%)	0 (0.0%)	0 (0.0%)	0.18	0 (0.0%)	1 (0.1%)	>0.99
Phosphorus, mg	974.3 (840.5, 1,219.6)	896.7 (706.5, 1,085.7)	711.3 (536.5, 925.4)	590.4 (428.0, 783.9)	<0.001	974.3 (841.3, 1,219.6)	661.6 (484.7, 893.1)	<0.001
Meeting RNI, n (%)	40 (81.6%)	81 (42.0%)	111 (33.0%)	140 (18.1%)	<0.001	42 (79.2%)	330 (25.4%)	<0.001

	High dairy consumer	Middle dairy consumer	Low dairy consumer	No dairy consumer	*p*-Value	Meeting daily dairy recommendations	Not meeting daily dairy recommendations	*p*-Value
**2-3 years**								
**Thailand, n**	376	170	96	82		376	348	
Energy, kcal	1,349.6 (1,136.9, 1,567.8)	1,167.1 (932.7, 1,417.2)	1,201.2 (956.7, 1,414.1)	1,261.1 (962.1, 1,456.7)	<0.001	1,349.6 (1,136.9, 1,567.8)	1,203.9 (954.3, 1,422.1)	<0.001
Meeting EER, n (%)	321 (85.4%)	119 (70.0%)	65 (67.7%)	58 (70.7%)	<0.001	321 (85.4%)	242 (69.5%)	<0.001
Protein, g	54.3 (44.4, 66.3)	46.9 (36.5, 56.4)	43.2 (33.9, 54.3)	39.9 (32.6, 56.5)	<0.001	54.3 (44.4, 66.3)	44 (35.2, 55.5)	<0.001
Meeting RNI, n (%)	375 (99.7%)	170 (100.0%)	96 (100.0%)	80 (97.6%)	0.087	375 (99.7%)	346 (99.4%)	0.61
Meeting EAR, n (%)	376 (100.0%)	170 (100.0%)	96 (100.0%)	80 (97.6%)	0.013	376 (100.0%)	346 (99.4%)	0.23
Carbohydrates, g	150.6 (120.2, 187.4)	136.5 (109.7, 177.5)	161.1 (113.2, 202.6)	175 (130.6, 201.0)	0.003	150.6 (120.2, 187.4)	153.4 (117.0, 188.8)	0.96
Fat, g	56.4 (43.7, 66.8)	42.8 (31.3, 55.8)	38.5 (30.2, 52.9)	42 (29.9, 57.3)	<0.001	56.4 (43.7, 66.8)	40.9 (30.5, 55.5)	<0.001
Fiber, g	5.43 (3.09, 8.28)	4.95 (3.23, 6.84)	4.22 (2.99, 6.04)	4.68 (3.03, 6.01)	0.010	5.43 (3.09, 8.28)	4.68 (3.08, 6.38)	0.003
Meeting RNI, n (%)	121 (32.2%)	30 (17.6%)	11 (11.5%)	11 (13.4%)	<0.001	121 (32.2%)	52 (14.9%)	<0.001
Vitamin A, μg RAE	512.7 (373.1, 758.1)	348.3 (225.3, 486.5)	298.2 (185.7, 419.3)	200.2 (116.0, 411.8)	<0.001	512.7 (373.1, 758.1)	299.1 (184.1, 455.1)	<0.001
Meeting RNI, n (%)	330 (87.8%)	96 (56.5%)	47 (49.0%)	30 (36.6%)	<0.001	330 (87.8%)	173 (49.7%)	<0.001
Meeting EAR, n (%)	356 (94.7%)	130 (76.5%)	67 (69.8%)	37 (45.1%)	<0.001	356 (94.7%)	234 (67.2%)	<0.001
Vitamin B_1, mg	0.77 (0.55, 1.26)	0.71 (0.47, 1.10)	0.69 (0.41, 0.95)	0.65 (0.39, 0.94)	<0.001	0.77 (0.55, 1.26)	0.69 (0.43, 1.00)	<0.001
Meeting RNI, n (%)	339 (90.2%)	141 (82.9%)	74 (77.1%)	61 (74.4%)	<0.001	339 (90.2%)	276 (79.3%)	<0.001
Meeting EAR, n (%)	311 (82.7%)	120 (70.6%)	61 (63.5%)	51 (62.2%)	<0.001	311 (82.7%)	232 (66.7%)	<0.001
Vitamin B_2, mg	1.81 (1.46, 2.23)	1.20 (1.00, 1.55)	1.00 (0.75, 1.38)	0.80 (0.55, 1.17)	<0.001	1.81 (1.46, 2.23)	1.08 (0.81, 1.46)	<0.001
Meeting RNI, n (%)	376 (100.0%)	165 (97.1%)	93 (96.9%)	64 (78.0%)	<0.001	376 (100.0%)	322 (92.5%)	<0.001
Meeting EAR, n (%)	376 (100.0%)	170 (100.0%)	95 (99.0%)	71 (86.6%)	<0.001	376 (100.0%)	336 (96.6%)	<0.001
Vitamin B_3, mg	7.6 (5.0, 11.9)	7.1 (5.1, 10.7)	7.7 (5.0, 12.1)	6.9 (4.7, 10.4)	0.60	7.6 (5.0, 11.9)	7.2 (5.0, 11.0)	0.47
Vitamin B_{12}, μg	2.56 (1.32, 4.09)	1.88 (1.27, 2.66)	1.71 (1.13, 2.49)	1.19 (0.59, 1.83)	<0.001	2.56 (1.32, 4.09)	1.68 (1.07, 2.55)	<0.001
Meeting RNI, n (%)	307 (81.6%)	147 (86.5%)	83 (86.5%)	49 (59.8%)	<0.001	307 (81.6%)	279 (80.2%)	0.61
Meeting EAR, n (%)	329 (87.5%)	157 (92.4%)	91 (94.8%)	56 (68.3%)	<0.001	329 (87.5%)	304 (87.4%)	0.95
Vitamin C, mg	22.9 (8.6, 54.4)	18.7 (9.6, 34.3)	19.5 (7.4, 34.2)	17.3 (6.8, 39.4)	0.10	22.9 (8.6, 54.4)	19 (8.5, 35.8)	0.013
Meeting RNI, n (%)	180 (47.9%)	66 (38.8%)	34 (35.4%)	35 (42.7%)	0.072	180 (47.9%)	135 (38.8%)	0.014
Meeting EAR, n (%)	210 (55.9%)	81 (47.6%)	47 (49.0%)	40 (48.8%)	0.24	210 (55.9%)	168 (48.3%)	0.041
Vitamin D, μg	7.7 (5.6, 10.1)	4.9 (3.8, 6.6)	3.8 (2.8, 4.8)	2.4 (1.1, 4.2)	<0.001	7.7 (5.6, 10.1)	4.2 (2.8, 5.8)	<0.001

Meeting RNI, n (%)	18 (4.8%)	1 (0.6%)	0 (0.0%)	2 (2.4%)	0.009	18 (4.8%)	3 (0.9%)	0.002
Meeting EAR, n (%)	97 (25.8%)	5 (2.9%)	1 (1.0%)	2 (2.4%)	<0.001	97 (25.8%)	8 (2.3%)	<0.001
Calcium, mg	858.1 (678.0, 1,076.7)	532.5 (439.0, 622.3)	378.9 (290.2, 492.7)	257.3 (176.8, 374.3)	<0.001	858.1 (678.0, 1,076.7)	440.1 (316.3, 580.8)	<0.001
Meeting RNI, n (%)	367 (97.6%)	101 (59.4%)	23 (24.0%)	14 (17.1%)	<0.001	367 (97.6%)	138 (39.7%)	<0.001
Iron, mg	5.8 (3.4, 11.0)	5 (3.6, 7.0)	4.9 (3.7, 6.6)	5.2 (3.8, 7.1)	0.013	5.8 (3.4, 11.0)	5 (3.7, 6.9)	0.001
Meeting RNI, n (%)	211 (56.1%)	82 (48.2%)	46 (47.9%)	43 (52.4%)	0.26	211 (56.1%)	171 (49.1%)	0.060
Meeting EAR, n (%)	234 (62.2%)	110 (64.7%)	58 (60.4%)	53 (64.6%)	0.88	234 (62.2%)	221 (63.5%)	0.72
Zinc, mg	3.86 (2.69, 5.94)	3.15 (2.38, 4.09)	3.09 (2.33, 3.91)	2.98 (2.16, 4.34)	<0.001	3.86 (2.69, 5.94)	3.10 (2.31, 4.02)	<0.001
Meeting RNI, n (%)	160 (42.6%)	34 (20.0%)	16 (16.7%)	18 (22.0%)	<0.001	160 (42.6%)	68 (19.5%)	<0.001
Meeting EAR, n (%)	197 (52.4%)	54 (31.8%)	28 (29.2%)	28 (34.1%)	<0.001	197 (52.4%)	110 (31.6%)	<0.001
Magnesium, mg	72.2 (47.5, 105.6)	64 (48.1, 87.1)	77.3 (62.3, 109.9)	68.2 (45.4, 99.8)	0.031	72.2 (47.5, 105.6)	69.2 (48.8, 96.9)	0.58
Meeting RNI, n (%)	236 (62.8%)	99 (58.2%)	75 (78.1%)	52 (63.4%)	0.012	236 (62.8%)	226 (64.9%)	0.54
Meeting EAR, n (%)	273 (72.6%)	120 (70.6%)	77 (80.2%)	59 (72.0%)	0.37	273 (72.6%)	256 (73.6%)	0.77
Sodium, mg	1,509.8 (1,064.8, 2,076.9)	1,580 (1,079.3, 2,168.7)	1,497.9 (969.1, 2,116.0)	1,402 (970.0, 2,088.4)	0.68	1,509.8 (1,064.8, 2,076.9)	1,507.1 (1,006.3, 2,128.9)	0.65
Meeting RNI, n (%)	373 (99.2%)	166 (97.6%)	95 (99.0%)	80 (97.6%)	0.29	373 (99.2%)	341 (98.0%)	0.21
Potassium, mg	1,163.9 (723.0, 1,674.5)	861.5 (640.7, 1,127.4)	821.7 (564.1, 1,072.8)	783 (540.8, 1,001.6)	<0.001	1,163.9 (723.0, 1,674.5)	842 (614.5, 1,105.7)	<0.001
Meeting RNI, n (%)	105 (27.9%)	13 (7.6%)	5 (5.2%)	6 (7.3%)	<0.001	105 (27.9%)	24 (6.9%)	<0.001
Phosphorus, mg	688.9 (443.5, 914.0)	474.1 (353.3, 628.1)	478.1 (378.1, 648.7)	425.8 (271.3, 542.4)	<0.001	688.9 (443.5, 914.0)	467.7 (348.4, 614.6)	<0.001
Meeting RNI, n (%)	273 (72.6%)	92 (54.1%)	52 (54.2%)	36 (43.9%)	<0.001	273 (72.6%)	180 (51.7%)	<0.001
Meeting EAR, n (%)	300 (79.8%)	123 (72.4%)	70 (72.9%)	51 (62.2%)	0.005	300 (79.8%)	244 (70.1%)	0.003
**4-6 years**								
**Thailand, n**	173	214	297	161		174	671	
Energy, kcal	1,587.4 (1,317.4, 1,798.5)	1,442.6 (1,197.6, 1,782.8)	1,420.4 (1,166.6, 1,787.2)	1,321 (988.6, 1,655.1)	<0.001	1,583.4 (1,317.4, 1,798.5)	1,402.9 (1,149.3, 1,757.8)	<0.001
Meeting EER, n (%)	124 (71.7%)	129 (60.3%)	167 (56.2%)	85 (52.8%)	0.002	125 (71.8%)	380 (56.6%)	<0.001
Protein, g	60.9 (50.8, 74.1)	53.5 (45.0, 67.6)	52.6 (41.6, 66.1)	45.8 (34.9, 59.2)	<0.001	60.8 (50.8, 74.1)	51.8 (40.6, 65.3)	<0.001
Meeting RNI, n (%)	173 (100.0%)	214 (100.0%)	297 (100.0%)	155 (96.3%)	<0.001	174 (100.0%)	665 (99.1%)	0.36
Meeting EAR, n (%)	173 (100.0%)	214 (100.0%)	297 (100.0%)	160 (99.4%)	0.19	174 (100.0%)	670 (99.9%)	>0.99
Carbohydrates, g	175.9 (142.0, 212.1)	184.7 (139.5, 226.7)	186.3 (145.7, 230.2)	177.7 (139.2, 231.5)	0.50	176 (142.0, 212.1)	181.4 (140.6, 228.8)	0.20

Fat, g	63.2 (51.7, 80.3)	51.7 (39.9, 66.3)	51.3 (38.0, 66.0)	41.6 (30.1, 57.2)	<0.001	63.2 (51.7, 80.3)	49.2 (37.4, 65.3)	<0.001
Fiber, g	6.39 (4.58, 8.63)	5.56 (4.14, 8.01)	5.28 (3.86, 7.00)	4.39 (2.73, 6.34)	<0.001	6.40 (4.58, 8.81)	5.24 (3.70, 7.08)	<0.001
Meeting RNI, n (%)	35 (20.2%)	30 (14.0%)	23 (7.7%)	11 (6.8%)	<0.001	35 (20.1%)	64 (9.5%)	<0.001
Vitamin A, μg RAE	510.4 (360.0, 650.0)	373.7 (256.1, 520.9)	297.7 (192.5, 439.5)	213.4 (124.1, 367.2)	<0.001	508.4 (340.6, 650.0)	307 (191.0, 464.7)	<0.001
Meeting RNI, n (%)	130 (75.1%)	119 (55.6%)	123 (41.4%)	45 (28.0%)	<0.001	130 (74.7%)	287 (42.8%)	<0.001
Meeting EAR, n (%)	133 (76.9%)	125 (58.4%)	128 (43.1%)	50 (31.1%)	<0.001	133 (76.4%)	303 (45.2%)	<0.001
Vitamin B_1, mg	0.91 (0.65, 1.38)	0.96 (0.64, 1.39)	0.82 (0.58, 1.29)	0.71 (0.43, 1.18)	<0.001	0.92 (0.65, 1.38)	0.85 (0.56, 1.30)	0.036
Meeting RNI, n (%)	149 (86.1%)	184 (86.0%)	238 (80.1%)	107 (66.5%)	<0.001	150 (86.2%)	528 (78.7%)	0.026
Meeting EAR, n (%)	137 (79.2%)	173 (80.8%)	214 (72.1%)	90 (55.9%)	<0.001	138 (79.3%)	476 (70.9%)	0.027
Vitamin B_2, mg	1.84 (1.52, 2.18)	1.37 (1.16, 1.76)	1.15 (0.89, 1.48)	0.81 (0.53, 1.15)	<0.001	1.83 (1.52, 2.18)	1.18 (0.87, 1.57)	<0.001
Meeting RNI, n (%)	173 (100.0%)	213 (99.5%)	289 (97.3%)	108 (67.1%)	<0.001	174 (100.0%)	609 (90.8%)	<0.001
Meeting EAR, n (%)	173 (100.0%)	214 (100.0%)	297 (100.0%)	125 (77.6%)	<0.001	174 (100.0%)	635 (94.6%)	0.002
Vitamin B_3, mg	9 (5.7, 12.7)	9.7 (6.7, 13.7)	10.1 (7.4, 14.0)	9.2 (6.2, 13.1)	0.10	9 (5.7, 12.8)	9.7 (6.8, 13.8)	0.073
Vitamin B_{12}, μg	2.78 (1.72, 4.22)	2.24 (1.55, 2.97)	1.96 (1.39, 2.79)	1.44 (0.76, 2.37)	<0.001	2.76 (1.71, 4.22)	1.92 (1.23, 2.80)	<0.001
Meeting RNI, n (%)	127 (73.4%)	140 (65.4%)	165 (55.6%)	58 (36.0%)	<0.001	127 (73.0%)	363 (54.1%)	<0.001
Meeting EAR, n (%)	154 (89.0%)	189 (88.3%)	257 (86.5%)	107 (66.5%)	<0.001	155 (89.1%)	552 (82.3%)	0.030
Vitamin C, mg	17.6 (8.3, 34.8)	21.2 (10.2, 34.7)	20.9 (11.2, 34.3)	22.5 (10.2, 44.0)	0.35	17.6 (8.3, 34.9)	21.6 (10.3, 35.6)	0.17
Meeting RNI, n (%)	45 (26.0%)	59 (27.6%)	83 (27.9%)	54 (33.5%)	0.45	46 (26.4%)	195 (29.1%)	0.49
Meeting EAR, n (%)	57 (32.9%)	85 (39.7%)	109 (36.7%)	67 (41.6%)	0.36	58 (33.3%)	260 (38.7%)	0.19
Vitamin D, μg	7.7 (5.8, 9.7)	5.4 (4.0, 6.8)	3.7 (2.8, 5.5)	2.3 (1.2, 4.0)	<0.001	7.7 (5.8, 9.7)	4 (2.6, 5.8)	<0.001
Meeting RNI, n (%)	7 (4.0%)	1 (0.5%)	2 (0.7%)	0 (0.0%)	0.004	7 (4.0%)	3 (0.4%)	<0.001
Meeting EAR, n (%)	38 (22.0%)	11 (5.1%)	13 (4.4%)	2 (1.2%)	<0.001	38 (21.8%)	26 (3.9%)	<0.001
Calcium, mg	826 (704.4, 1,007.3)	554.3 (483.8, 669.3)	405.2 (315.6, 536.0)	200.3 (116.2, 364.4)	<0.001	824.6 (704.4, 1,007.3)	447.1 (304.6, 562.7)	<0.001
Meeting RNI, n (%)	91 (52.6%)	15 (7.0%)	10 (3.4%)	7 (4.3%)	<0.001	91 (52.3%)	32 (4.8%)	<0.001
Iron, mg	5.7 (4.2, 8.7)	5.8 (4.2, 8.2)	6.1 (4.6, 8.5)	6 (4.1, 7.6)	0.51	5.7 (4.2, 8.7)	6 (4.4, 8.2)	0.86
Meeting RNI, n (%)	74 (42.8%)	98 (45.8%)	147 (49.5%)	75 (46.6%)	0.56	74 (42.5%)	320 (47.7%)	0.22
Meeting EAR, n (%)	104 (60.1%)	136 (63.6%)	206 (69.4%)	109 (67.7%)	0.18	105 (60.3%)	450 (67.1%)	0.10
Zinc, mg	4.06 (3.02, 5.34)	3.87 (3.06, 5.14)	4.01 (3.19, 5.03)	3.69 (2.56, 4.80)	0.066	4.05 (3.01, 5.34)	3.94 (3.00, 4.99)	0.59
Meeting RNI, n (%)	42 (24.3%)	46 (21.5%)	54 (18.2%)	30 (18.6%)	0.40	42 (24.1%)	130 (19.4%)	0.16
Meeting EAR, n (%)	60 (34.7%)	65 (30.4%)	100 (33.7%)	49 (30.4%)	0.73	60 (34.5%)	214 (31.9%)	0.52
Magnesium, mg	85.2 (59.3, 109.5)	85.1 (64.1, 114.9)	89.7 (66.7, 126.2)	87 (54.6, 129.9)	0.087	85.8 (59.3, 110.4)	88.2 (64.0, 124.0)	0.082
Meeting RNI, n (%)	80 (46.2%)	104 (48.6%)	167 (56.2%)	82 (50.9%)	0.15	81 (46.6%)	352 (52.5%)	0.16

Meeting EAR, n (%)	110 (63.6%)	142 (66.4%)	201 (67.7%)	97 (60.2%)	0.41	111 (63.8%)	439 (65.4%)	0.69
Sodium, mg	1,979.6 (1,312.7, 2,574.7)	1,926.9 (1,393.5, 2,508.2)	1,968.7 (1,446.4, 2,602.6)	1,816 (1,259.3, 2,562.3)	0.28	1,983.4 (1,312.7, 2,574.7)	1,908.8 (1,378.6, 2,562.3)	0.94
Meeting RNI, n (%)	171 (98.8%)	211 (98.6%)	296 (99.7%)	156 (96.9%)	0.089	172 (98.9%)	662 (98.7%)	>0.99
Potassium, mg	1,162.1 (808.6, 1,513.1)	1,065.1 (775.4, 1,407.1)	1,028.4 (757.3, 1,354.5)	899.3 (614.0, 1,269.7)	<0.001	1,159.9 (808.6, 1,513.1)	1,004.3 (726.0, 1,345.0)	0.003
Meeting RNI, n (%)	13 (7.5%)	5 (2.3%)	7 (2.4%)	2 (1.2%)	0.003	13 (7.5%)	14 (2.1%)	<0.001
Phosphorus, mg	681.9 (487.4, 921.1)	615.5 (467.6, 796.6)	607.6 (457.3, 776.4)	477.2 (326.8, 643.4)	<0.001	682.8 (487.4, 921.1)	577.7 (421.3, 766.5)	<0.001
Meeting RNI, n (%)	128 (74.0%)	149 (69.6%)	202 (68.0%)	76 (47.2%)	<0.001	129 (74.1%)	426 (63.5%)	0.008
Meeting EAR, n (%)	150 (86.7%)	178 (83.2%)	247 (83.2%)	102 (63.4%)	<0.001	151 (86.8%)	526 (78.4%)	0.013
**7-12 years**								
**Thailand, n**	70	143	403	461		70	1007	
Energy, kcal	1,815.7 (1,599.2, 2,372.0)	1,812.7 (1,437.8, 2,241.7)	1,694.3 (1,343.9, 2,041.5)	1,602 (1,252.5, 1,959.3)	<0.001	1,815.7 (1,599.2, 2,372.0)	1,660.8 (1,323.3, 2,030.0)	0.001
Meeting EER, n (%)	55 (78.6%)	98 (68.5%)	230 (57.1%)	213 (46.2%)	<0.001	55 (78.6%)	541 (53.7%)	<0.001
Protein, g	69.5 (55.9, 89.9)	70.6 (54.9, 88.4)	63.5 (48.9, 80.6)	56.1 (43.0, 75.3)	<0.001	69.5 (55.9, 89.9)	60.7 (46.9, 79.0)	<0.001
Meeting RNI, n (%)	68 (97.1%)	141 (98.6%)	384 (95.3%)	400 (86.8%)	<0.001	68 (97.1%)	925 (91.9%)	0.11
Meeting EAR, n (%)	69 (98.6%)	143 (100.0%)	400 (99.3%)	446 (96.7%)	0.012	69 (98.6%)	989 (98.2%)	>0.99
Carbohydrates, g	231.8 (190.5, 285.3)	224.1 (176.1, 272.7)	222.1 (174.2, 274.1)	219.6 (173.9, 272.6)	0.51	231.8 (190.5, 285.3)	221.3 (174.2, 272.9)	0.18
Fat, g	68.2 (50.5, 97.6)	67.7 (52.2, 87.3)	55.4 (41.3, 74.3)	49.9 (32.7, 70.1)	<0.001	68.2 (50.5, 97.6)	54.5 (39.6, 75.3)	<0.001
Fiber, g	7.74 (5.93, 9.90)	7.07 (5.33, 9.79)	6.58 (4.67, 9.21)	5.69 (3.96, 8.23)	<0.001	7.74 (5.93, 9.90)	6.23 (4.44, 8.70)	0.002
Meeting RNI, n (%)	5 (7.1%)	7 (4.9%)	19 (4.7%)	17 (3.7%)	0.51	5 (7.1%)	43 (4.3%)	0.23
Vitamin A, μg RAE	445.5 (310.3, 665.4)	419.1 (260.1, 616.6)	268.8 (159.3, 398.1)	222.6 (116.8, 371.2)	<0.001	445.5 (310.3, 665.4)	261.6 (150.1, 420.4)	<0.001
Meeting RNI, n (%)	38 (54.3%)	60 (42.0%)	84 (20.8%)	72 (15.6%)	<0.001	38 (54.3%)	216 (21.4%)	<0.001
Meeting EAR, n (%)	46 (65.7%)	76 (53.1%)	112 (27.8%)	112 (24.3%)	<0.001	46 (65.7%)	300 (29.8%)	<0.001
Vitamin B_1, mg	1.20 (0.71, 1.88)	1.07 (0.74, 1.52)	0.84 (0.58, 1.33)	0.78 (0.50, 1.19)	<0.001	1.20 (0.71, 1.88)	0.84 (0.56, 1.30)	<0.001
Meeting RNI, n (%)	59 (84.3%)	122 (85.3%)	287 (71.2%)	292 (63.3%)	<0.001	59 (84.3%)	701 (69.6%)	0.009
Meeting EAR, n (%)	54 (77.1%)	102 (71.3%)	228 (56.6%)	225 (48.8%)	<0.001	54 (77.1%)	555 (55.1%)	<0.001
Vitamin B_2, mg	1.81 (1.54, 2.19)	1.49 (1.19, 1.83)	1.10 (0.88, 1.45)	0.77 (0.54, 1.14)	<0.001	1.81 (1.54, 2.19)	1.02 (0.73, 1.42)	<0.001
Meeting RNI, n (%)	70 (100.0%)	137 (95.8%)	330 (81.9%)	210 (45.6%)	<0.001	70 (100.0%)	677 (67.2%)	<0.001
Meeting EAR, n (%)	70 (100.0%)	140 (97.9%)	370 (91.8%)	260 (56.4%)	<0.001	70 (100.0%)	770 (76.5%)	<0.001

Vitamin B_3, mg	11.5 (8.6, 16.5)	12.8 (8.4, 17.1)	11.3 (7.8, 15.5)	10.9 (7.7, 14.4)	0.019	11.5 (8.6, 16.5)	11.3 (7.8, 15.3)	0.27
Vitamin B_{12}, µg	2.91 (2.36, 3.81)	2.66 (1.67, 3.71)	1.95 (1.32, 3.00)	1.56 (0.89, 2.61)	<0.001	2.91 (2.36, 3.81)	1.87 (1.10, 2.95)	<0.001
Meeting RNI, n (%)	63 (90.0%)	107 (74.8%)	225 (55.8%)	195 (42.3%)	<0.001	63 (90.0%)	527 (52.3%)	<0.001
Meeting EAR, n (%)	66 (94.3%)	117 (81.8%)	292 (72.5%)	264 (57.3%)	<0.001	66 (94.3%)	673 (66.8%)	<0.001
Vitamin C, mg	21 (13.7, 45.0)	21.7 (13.0, 41.8)	23.6 (12.5, 38.9)	21.2 (9.9, 39.6)	0.24	21 (13.7, 45.0)	22.3 (11.0, 39.6)	0.38
Meeting RNI, n (%)	19 (27.1%)	28 (19.6%)	87 (21.6%)	88 (19.1%)	0.42	19 (27.1%)	203 (20.2%)	0.16
Meeting EAR, n (%)	19 (27.1%)	35 (24.5%)	101 (25.1%)	98 (21.3%)	0.49	19 (27.1%)	234 (23.2%)	0.46
Vitamin D, µg	6.8 (5.4, 8.4)	5.7 (4.0, 7.5)	3.7 (2.5, 5.4)	2 (1.0, 3.6)	<0.001	6.8 (5.4, 8.4)	3.3 (1.9, 5.1)	<0.001
Meeting RNI, n (%)	0 (0.0%)	6 (4.2%)	4 (1.0%)	3 (0.7%)	0.023	0 (0.0%)	13 (1.3%)	>0.99
Meeting EAR, n (%)	9 (12.9%)	13 (9.1%)	15 (3.7%)	8 (1.7%)	<0.001	9 (12.9%)	36 (3.6%)	0.002
Calcium, mg	777.8 (681.9, 949.4)	586.1 (493.6, 699.0)	397.8 (317.0, 506.6)	202 (123.8, 325.9)	<0.001	777.8 (681.9, 949.4)	345.8 (219.8, 502.0)	<0.001
Meeting RNI, n (%)	21 (30.0%)	9 (6.3%)	4 (1.0%)	2 (0.4%)	<0.001	21 (30.0%)	15 (1.5%)	<0.001
Iron, mg	7.7 (5.1, 10.9)	7.5 (5.2, 10.1)	7.1 (5.2, 9.6)	6.8 (4.9, 9.3)	0.25	7.7 (5.1, 10.9)	7 (5.1, 9.5)	0.20
Meeting RNI, n (%)	32 (45.7%)	61 (42.7%)	151 (37.5%)	132 (28.6%)	<0.001	32 (45.7%)	344 (34.2%)	0.050
Meeting EAR, n (%)	29 (41.4%)	54 (37.8%)	147 (36.5%)	138 (29.9%)	0.069	29 (41.4%)	339 (33.7%)	0.19
Zinc, mg	4.58 (3.55, 6.46)	4.93 (3.77, 6.72)	4.77 (3.48, 6.23)	4.50 (3.32, 5.92)	0.095	4.58 (3.55, 6.46)	4.69 (3.43, 6.13)	0.63
Meeting RNI, n (%)	7 (10.0%)	18 (12.6%)	41 (10.2%)	41 (8.9%)	0.63	7 (10.0%)	100 (9.9%)	0.99
Meeting EAR, n (%)	17 (24.3%)	35 (24.5%)	84 (20.8%)	70 (15.2%)	0.026	17 (24.3%)	189 (18.8%)	0.26
Magnesium, mg	109 (86.2, 143.1)	114.1 (78.2, 140.3)	103.7 (75.1, 138.2)	97.2 (66.8, 136.6)	0.004	109 (86.2, 143.1)	102.2 (71.8, 138.2)	0.17
Meeting RNI, n (%)	15 (21.4%)	31 (21.7%)	95 (23.6%)	69 (15.0%)	0.012	15 (21.4%)	195 (19.4%)	0.67
Meeting EAR, n (%)	29 (41.4%)	54 (37.8%)	138 (34.2%)	132 (28.6%)	0.047	29 (41.4%)	324 (32.2%)	0.11
Sodium, mg	2,455.8 (1,845.1, 3,613.9)	2,609.7 (1,811.0, 3,469.7)	2,301.2 (1,665.5, 3,287.1)	2,346.6 (1,666.8, 3,412.2)	0.26	2,455.8 (1,845.1, 3,613.9)	2,354.3 (1,685.1, 3,360.4)	0.45
Meeting RNI, n (%)	69 (98.6%)	140 (97.9%)	396 (98.3%)	452 (98.0%)	0.98	69 (98.6%)	988 (98.1%)	>0.99
Potassium, mg	1,330.2 (1,049.4, 1,738.3)	1,276 (923.6, 1,640.2)	1,183.7 (902.7, 1,565.8)	1,062.4 (768.9, 1,452.4)	<0.001	1,330.2 (1,049.4, 1,738.3)	1,126.3 (839.1, 1,528.7)	<0.001
Meeting RNI, n (%)	4 (5.7%)	3 (2.1%)	4 (1.0%)	11 (2.4%)	0.063	4 (5.7%)	18 (1.8%)	0.049
Phosphorus, mg	864.1 (689.8, 1,056.5)	745.2 (545.2, 972.5)	661.6 (482.1, 860.2)	550.2 (395.3, 778.0)	<0.001	864.1 (689.8, 1,056.5)	624.7 (446.9, 845.2)	<0.001
Meeting RNI, n (%)	41 (58.6%)	66 (46.2%)	138 (34.2%)	112 (24.3%)	<0.001	41 (58.6%)	316 (31.4%)	<0.001
Meeting EAR, n (%)	54 (77.1%)	91 (63.6%)	194 (48.1%)	165 (35.8%)	<0.001	54 (77.1%)	450 (44.7%)	<0.001

	High dairy consumer	Middle dairy consumer	Low dairy consumer	No dairy consumer	*p*-Value	Meeting daily dairy recommendations	Not meeting daily dairy recommendations	*p*-Value
**2-3 years**								
**Vietnam, n**	273	151	155	109		137	551	
Energy, kcal	1,027 (848.8, 1,249.2)	888.7 (763.3, 1,056.2)	817.7 (651.8, 963.0)	812.8 (679.3, 1,025.7)	<0.001	1,119.3 (938.6, 1,315.0)	878.5 (713.2, 1,037.9)	<0.001
Meeting EER, n (%)	119 (43.6%)	35 (23.2%)	22 (14.2%)	22 (20.2%)	<0.001	71 (51.8%)	127 (23.0%)	<0.001
Protein, g	43.6 (33.3, 54.5)	38 (30.9, 48.5)	33.2 (25.5, 43.1)	31.6 (25.2, 40.1)	<0.001	47.6 (36.9, 58.2)	36.1 (28.1, 45.4)	<0.001
Meeting RNI, n (%)	261 (95.6%)	147 (97.4%)	129 (83.2%)	90 (82.6%)	<0.001	133 (97.1%)	494 (89.7%)	0.006
Carbohydrates, g	145 (113.9, 174.6)	130.2 (107.2, 153.5)	125 (94.7, 149.2)	137.1 (107.8, 174.2)	<0.001	156.9 (125.6, 182.7)	131 (103.0, 158.9)	<0.001
Meeting RNI, n (%)	112 (41.0%)	41 (27.2%)	30 (19.4%)	38 (34.9%)	<0.001	70 (51.1%)	151 (27.4%)	<0.001
Fat, g	29.1 (22.9, 38.8)	23.1 (18.1, 31.5)	18.6 (14.1, 25.5)	15.5 (11.4, 21.6)	<0.001	31.7 (26.2, 41.8)	21.5 (15.5, 29.3)	<0.001
Meeting RNI, n (%)	96 (35.2%)	32 (21.2%)	15 (9.7%)	9 (8.3%)	<0.001	60 (43.8%)	92 (16.7%)	<0.001
Vitamin A, µg RAE	420.4 (311.8, 565.6)	295.1 (209.0, 468.0)	229.6 (136.2, 410.0)	319.9 (100.3, 738.0)	<0.001	474.7 (385.8, 622.3)	300.3 (191.5, 495.4)	<0.001
Meeting RNI, n (%)	137 (50.2%)	47 (31.1%)	41 (26.5%)	48 (44.0%)	<0.001	88 (64.2%)	185 (33.6%)	<0.001
Meeting EAR, n (%)	209 (76.6%)	79 (52.3%)	58 (37.4%)	58 (53.2%)	<0.001	120 (87.6%)	284 (51.5%)	<0.001
Vitamin B_{12}, µg	2.29 (1.72, 3.33)	1.50 (1.24, 2.02)	1.12 (0.79, 1.51)	0.80 (0.65, 1.37)	<0.001	2.68 (2.04, 3.72)	1.45 (0.95, 2.02)	<0.001
Meeting RNI, n (%)	269 (98.5%)	140 (92.7%)	98 (63.2%)	44 (40.4%)	<0.001	137 (100.0%)	414 (75.1%)	<0.001
Meeting EAR, n (%)	270 (98.9%)	141 (93.4%)	112 (72.3%)	49 (45.0%)	<0.001	137 (100.0%)	435 (78.9%)	<0.001
Vitamin C, mg	28.2 (12.8, 56.1)	27.6 (17.1, 47.3)	22.8 (11.4, 46.0)	17.3 (7.8, 29.8)	<0.001	28.5 (11.7, 67.7)	23.7 (11.6, 43.6)	0.034
Meeting RNI, n (%)	109 (39.9%)	49 (32.5%)	50 (32.3%)	22 (20.2%)	0.003	59 (43.1%)	171 (31.0%)	0.008
Meeting EAR, n (%)	126 (46.2%)	64 (42.4%)	57 (36.8%)	25 (22.9%)	<0.001	63 (46.0%)	209 (37.9%)	0.084
Vitamin D, µg	6 (4.3, 10.2)	3.2 (2.5, 5.3)	2 (1.4, 4.8)	0.6 (0.3, 1.3)	<0.001	7.6 (5.5, 12.0)	2.9 (1.4, 5.5)	<0.001
Meeting RNI, n (%)	37 (13.6%)	5 (3.3%)	2 (1.3%)	4 (3.7%)	<0.001	27 (19.7%)	21 (3.8%)	<0.001
Calcium, mg	620.8 (528.3, 758.9)	428.9 (379.7, 484.3)	318.9 (233.8, 375.0)	187.2 (142.6, 238.8)	<0.001	743.2 (627.3, 880.2)	394.8 (258.0, 505.3)	<0.001
Meeting RNI, n (%)	191 (70.0%)	21 (13.9%)	6 (3.9%)	7 (6.4%)	<0.001	125 (91.2%)	100 (18.1%)	<0.001
Iron, mg	5.9 (4.1, 8.2)	5.7 (3.9, 7.2)	5 (3.3, 6.4)	5.8 (4.6, 6.7)	<0.001	6.5 (4.3, 9.0)	5.4 (3.9, 7.0)	<0.001
Meeting RNI, n (%)	156 (57.1%)	89 (58.9%)	65 (41.9%)	61 (56.0%)	0.008	86 (62.8%)	285 (51.7%)	0.020
Zinc, mg	6.09 (4.73, 7.69)	5.24 (4.05, 6.64)	4.59 (3.48, 5.76)	4.44 (3.45, 5.78)	<0.001	6.57 (5.14, 8.26)	4.95 (3.86, 6.23)	<0.001
Meeting RNI, n (%)	214 (78.4%)	105 (69.5%)	86 (55.5%)	56 (51.4%)	<0.001	119 (86.9%)	342 (62.1%)	<0.001

**4-6 years**								
**Vietnam, n**	150	107	227	276		60	700	
Energy, kcal	1,333.4 (1,091.8, 1,544.5)	1,080.3 (893.6, 1,355.2)	1,051 (874.7, 1,297.0)	957.9 (742.3, 1,227.7)	<0.001	1,365.3 (1,141.1, 1,647.9)	1,041.2 (840.9, 1,310.4)	<0.001
Meeting EER, n (%)	75 (50.0%)	23 (21.5%)	39 (17.2%)	38 (13.8%)	<0.001	34 (56.7%)	141 (20.1%)	<0.001
Protein, g	55.8 (45.9, 69.9)	48.3 (39.2, 62.2)	44.9 (36.7, 53.9)	38.1 (30.2, 51.6)	<0.001	56.3 (48.5, 74.3)	44.6 (33.9, 56.8)	<0.001
Meeting RNI, n (%)	147 (98.0%)	99 (92.5%)	201 (88.5%)	207 (75.0%)	<0.001	58 (96.7%)	596 (85.1%)	0.013
Carbohydrates, g	185.1 (151.9, 214.2)	157.8 (121.2, 195.6)	161.5 (128.6, 198.9)	150.6 (115.5, 193.0)	<0.001	195.8 (153.8, 227.5)	159.3 (124.7, 198.1)	<0.001
Meeting RNI, n (%)	70 (46.7%)	29 (27.1%)	63 (27.8%)	62 (22.5%)	<0.001	36 (60.0%)	188 (26.9%)	<0.001
Fat, g	38.2 (29.0, 47.5)	29.2 (22.2, 38.9)	25.3 (17.4, 34.6)	19.4 (12.6, 27.8)	<0.001	39 (32.6, 48.0)	24.9 (17.0, 35.6)	<0.001
Meeting RNI, n (%)	90 (60.0%)	40 (37.4%)	60 (26.4%)	44 (15.9%)	<0.001	43 (71.7%)	191 (27.3%)	<0.001
Vitamin A, μg RAE	420.6 (314.7, 572.0)	302 (203.1, 446.8)	247.3 (146.3, 417.3)	212.5 (82.9, 418.3)	<0.001	501.8 (397.3, 666.4)	262.8 (148.6, 446.7)	<0.001
Meeting RNI, n (%)	70 (46.7%)	31 (29.0%)	54 (23.8%)	63 (22.8%)	<0.001	36 (60.0%)	182 (26.0%)	<0.001
Meeting EAR, n (%)	107 (71.3%)	53 (49.5%)	89 (39.2%)	94 (34.1%)	<0.001	50 (83.3%)	293 (41.9%)	<0.001
Vitamin B_{12}, μg	2.62 (2.16, 3.46)	1.89 (1.38, 2.73)	1.41 (0.96, 2.20)	0.84 (0.46, 1.89)	<0.001	3.11 (2.56, 4.79)	1.52 (0.83, 2.39)	<0.001
Meeting RNI, n (%)	148 (98.7%)	96 (89.7%)	147 (64.8%)	120 (43.5%)	<0.001	60 (100.0%)	451 (64.4%)	<0.001
Meeting EAR, n (%)	148 (98.7%)	98 (91.6%)	165 (72.7%)	130 (47.1%)	<0.001	60 (100.0%)	481 (68.7%)	<0.001
Vitamin C, mg	26.2 (10.0, 53.6)	26.1 (13.1, 49.9)	24.9 (12.4, 48.9)	15.3 (5.0, 40.7)	<0.001	25.3 (7.3, 54.1)	23.4 (8.7, 45.6)	0.57
Meeting RNI, n (%)	45 (30.0%)	33 (30.8%)	60 (26.4%)	59 (21.4%)	0.13	20 (33.3%)	177 (25.3%)	0.17
Meeting EAR, n (%)	54 (36.0%)	37 (34.6%)	71 (31.3%)	71 (25.7%)	0.11	21 (35.0%)	212 (30.3%)	0.45
Vitamin D, μg	6.4 (4.3, 13.8)	3.8 (2.7, 5.3)	2.5 (1.6, 4.8)	0.8 (0.4, 2.1)	<0.001	9 (5.7, 19.1)	2.5 (0.9, 4.9)	<0.001
Meeting RNI, n (%)	30 (20.0%)	5 (4.7%)	6 (2.6%)	9 (3.3%)	<0.001	21 (35.0%)	29 (4.1%)	<0.001
Calcium, mg	656.1 (557.4, 769.4)	451.2 (388.5, 543.5)	353.5 (274.0, 426.1)	186.4 (131.7, 255.9)	<0.001	766.7 (695.3, 884.7)	332.4 (203.5, 478.1)	<0.001
Meeting RNI, n (%)	87 (58.0%)	17 (15.9%)	12 (5.3%)	4 (1.4%)	<0.001	57 (95.0%)	63 (9.0%)	<0.001
Iron, mg	6.8 (5.2, 8.8)	6.8 (5.1, 8.2)	6.3 (4.9, 8.1)	5.8 (4.2, 7.6)	<0.001	7.4 (5.2, 10.3)	6.2 (4.7, 8.0)	0.004
Meeting RNI, n (%)	96 (64.0%)	62 (57.9%)	117 (51.5%)	106 (38.4%)	<0.001	40 (66.7%)	341 (48.7%)	0.008
Zinc, mg	7.40 (5.85, 8.42)	6.37 (4.82, 7.73)	5.88 (4.61, 7.00)	4.96 (3.86, 6.79)	<0.001	7.80 (6.18, 9.82)	5.74 (4.42, 7.23)	<0.001
Meeting RNI, n (%)	128 (85.3%)	76 (71.0%)	146 (64.3%)	127 (46.0%)	<0.001	53 (88.3%)	424 (60.6%)	<0.001
**7-12 years**								
**Vietnam, n**	133	99	395	914		42	1499	

Energy, kcal	1,375 (1,143.0, 1,739.4)	1,267.4 (961.7, 1,533.1)	1,299.1 (1,051.4, 1,542.9)	1,161 (917.0, 1,477.8)	<0.001	1,580.2 (1,186.4, 1,875.4)	1,221.8 (969.3, 1,512.3)	<0.001
Meeting EER, n (%)	40 (30.1%)	12 (12.1%)	57 (14.4%)	82 (9.0%)	<0.001	20 (47.6%)	171 (11.4%)	<0.001
Protein, g	61.8 (49.1, 82.8)	56.8 (41.4, 74.0)	58.1 (43.4, 71.3)	49.9 (37.2, 64.3)	<0.001	70.2 (51.8, 86.2)	53 (39.9, 67.7)	<0.001
Meeting RNI, n (%)	122 (91.7%)	79 (79.8%)	331 (83.8%)	630 (68.9%)	<0.001	40 (95.2%)	1,122 (74.8%)	0.002
Carbohydrates, g	192.6 (154.7, 251.2)	188.5 (131.5, 223.9)	186.2 (155.6, 228.4)	181.5 (140.8, 230.1)	0.036	202.3 (154.1, 280.3)	183.1 (144.4, 230.0)	0.046
Meeting RNI, n (%)	45 (33.8%)	22 (22.2%)	88 (22.3%)	198 (21.7%)	0.019	18 (42.9%)	335 (22.3%)	0.002
Fat, g	39.2 (31.2, 48.4)	29.9 (20.4, 43.1)	31.1 (23.4, 43.4)	23.6 (15.5, 34.4)	<0.001	41.7 (34.9, 54.6)	27.5 (18.0, 38.8)	<0.001
Meeting RNI, n (%)	73 (54.9%)	28 (28.3%)	131 (33.2%)	167 (18.3%)	<0.001	30 (71.4%)	369 (24.6%)	<0.001
Vitamin A, μg RAE	409.2 (306.6, 609.7)	336.6 (228.7, 540.9)	251.2 (169.2, 455.9)	208 (82.5, 456.2)	<0.001	465.6 (364.0, 749.7)	246.1 (127.3, 470.9)	<0.001
Meeting RNI, n (%)	52 (39.1%)	26 (26.3%)	89 (22.5%)	203 (22.2%)	<0.001	20 (47.6%)	350 (23.3%)	<0.001
Meeting EAR, n (%)	91 (68.4%)	45 (45.5%)	137 (34.7%)	277 (30.3%)	<0.001	37 (88.1%)	513 (34.2%)	<0.001
Vitamin B_{12}, μg	3.04 (2.30, 4.59)	2.13 (1.44, 3.31)	1.78 (1.22, 2.73)	1.07 (0.48, 2.12)	<0.001	3.18 (2.73, 4.48)	1.47 (0.76, 2.59)	<0.001
Meeting RNI, n (%)	133 (100.0%)	69 (69.7%)	243 (61.5%)	337 (36.9%)	<0.001	42 (100.0%)	740 (49.4%)	<0.001
Meeting EAR, n (%)	133 (100.0%)	81 (81.8%)	285 (72.2%)	397 (43.4%)	<0.001	42 (100.0%)	854 (57.0%)	<0.001
Vitamin C, mg	27.9 (9.1, 58.1)	28.8 (16.5, 80.1)	26.1 (12.6, 57.7)	22.1 (8.5, 55.3)	0.019	36.1 (11.4, 55.8)	24.3 (10.0, 58.3)	0.54
Meeting RNI, n (%)	32 (24.1%)	32 (32.3%)	90 (22.8%)	207 (22.6%)	0.19	9 (21.4%)	352 (23.5%)	0.76
Meeting EAR, n (%)	43 (32.3%)	38 (38.4%)	109 (27.6%)	238 (26.0%)	0.040	14 (33.3%)	414 (27.6%)	0.41
Vitamin D, μg	6.2 (4.3, 14.4)	4 (2.6, 8.1)	2.6 (1.8, 5.4)	1 (0.4, 2.9)	<0.001	6.9 (5.5, 18.4)	1.9 (0.6, 4.6)	<0.001
Meeting RNI, n (%)	29 (21.8%)	7 (7.1%)	19 (4.8%)	41 (4.5%)	<0.001	12 (28.6%)	84 (5.6%)	<0.001
Calcium, mg	661.8 (571.0, 865.0)	481 (386.4, 605.3)	397.5 (333.3, 492.6)	219.6 (156.7, 307.7)	<0.001	862.3 (682.3, 993.6)	307.3 (197.2, 434.9)	<0.001
Meeting RNI, n (%)	52 (39.1%)	13 (13.1%)	19 (4.8%)	18 (2.0%)	<0.001	33 (78.6%)	69 (4.6%)	<0.001
Iron, mg	7.6 (5.7, 10.1)	7.5 (5.7, 10.1)	7.7 (5.8, 9.6)	7.4 (5.6, 9.5)	0.36	8.1 (5.8, 11.1)	7.4 (5.7, 9.5)	0.19
Meeting RNI, n (%)	78 (58.6%)	57 (57.6%)	229 (58.0%)	474 (51.9%)	0.12	28 (66.7%)	810 (54.0%)	0.11
Zinc, mg	7.68 (6.25, 9.96)	6.77 (5.21, 9.24)	7.04 (5.51, 8.78)	6.32 (4.90, 8.30)	<0.001	8.10 (6.74, 10.55)	6.59 (5.11, 8.49)	<0.001
Meeting RNI, n (%)	105 (78.9%)	58 (58.6%)	247 (62.5%)	466 (51.0%)	<0.001	37 (88.1%)	839 (56.0%)	<0.001

Data are presented as medians (IQR), frequencies and proportions (%). Data presented as medians (IQR) across the different dairy consuming groups were analyzed using Kruskal-Wallis tests. Data presented as medians (IQR) across groups for meeting versus not meeting daily dairy recommendations were analyzed using Wilcoxon's rank sum tests.
