# Supplementary material for: Associations Between Dairy Consumption and Nutrient Intake in Southeast Asian Children: Findings from the South East Asian Nutrition Surveys II (SEANUTS II)
Source: Nutrients. 2025 Nov 28;17(23):3740. doi: 10.3390/nu17233740 (PMC12694304; doi:10.3390/nu17233740)
Supplement: Supplementary file 1 [file nutrients-17-03740-s001.zip › supplementary table S1.pdf]

Supplementary table S1. Median food intake, among high, middle, low, and non-dairy consumers as well as among children meeting and not meeting the daily dairy recommendations, stratified by age.

Food intake (servings/day)	High dairy consumer	Middle dairy consumer	Low dairy consumer	No dairy consumer	*p*-Value	Meeting daily dairy recommendation	Not meeting daily dairy recommendation	*p*-Value
**2-3 years**								
**Indonesia, n**	170	111	116	232		281	348	
Cereals & grains	1.8 (1.1, 2.6)	1.9 (1.2, 3.0)	1.6 (1.2, 2.9)	2.2 (1.5, 3.0)	0.004	1.8 (1.1, 2.8)	2.0 (1.5, 3.0)	0.023
Fruits	0.0 (0.0, 0.8)	0.0 (0.0, 0.6)	0.0 (0.0, 1.1)	0.0 (0.0, 0.8)	0.45	0.0 (0.0, 0.7)	0.0 (0.0, 0.9)	0.15
Vegetables	0.2 (0.0, 0.5)	0.2 (0.0, 0.5)	0.2 (0.0, 0.5)	0.2 (0.0, 0.6)	0.59	0.2 (0.0, 0.5)	0.2 (0.0, 0.6)	0.78
Meat & proteinaceous foods	1.9 (1.0, 2.8)	2.1 (1.2, 3.1)	2.4 (1.3, 3.2)	1.8 (1.1, 3.0)	0.076	1.9 (1.1, 2.9)	2.0 (1.2, 3.1)	0.44
Extra foods	4.7 (2.4, 7.5)	5.4 (3.3, 7.6)	5.7 (3.4, 8.3)	6.0 (4.0, 9.0)	0.002	5.0 (2.9, 7.6)	5.9 (3.8, 8.9)	<0.001
**4-6 years**								
**Indonesia, n**	91	54	96	281		145	377	
Cereals & grains	2.4 (1.6, 3.3)	2.0 (1.5, 3.5)	2.5 (1.5, 3.6)	2.5 (1.5, 3.5)	0.87	2.3 (1.6, 3.3)	2.5 (1.5, 3.6)	0.54
Fruits	0.0 (0.0, 0.6)	0.0 (0.0, 1.1)	0.0 (0.0, 0.9)	0.0 (0.0, 1.0)	0.97	0.0 (0.0, 0.8)	0.0 (0.0, 1.0)	0.78
Vegetables	0.2 (0.0, 0.4)	0.1 (0.0, 0.5)	0.2 (0.0, 0.5)	0.2 (0.0, 0.6)	0.39	0.2 (0.0, 0.5)	0.2 (0.0, 0.6)	0.14
Meat & proteinaceous foods	2.6 (1.8, 4.0)	2.3 (1.5, 3.5)	2.4 (1.6, 3.6)	2.3 (1.4, 3.3)	0.28	2.5 (1.7, 3.7)	2.3 (1.4, 3.4)	0.14
Extra foods	6.8(4.0, 10.5)	6.5 (4.4, 9.3)	7.2(4.9, 10.0)	7.1(4.7, 10.2)	0.70	6.7 (4.2, 9.8)	7.1 (4.7, 10.1)	0.24
**7-12 years**								
**Indonesia, n**	25	77	125	838		102	963	
Cereals & grains	3.5 (2.1, 5.1)	3.9 (2.5, 6.0)	3.5 (2.0, 4.7)	3.6 (2.4, 5.4)	0.075	3.8 (2.5, 5.4)	3.6 (2.3, 5.3)	0.39
Fruits	0.0 (0.0, 1.4)	0.0 (0.0, 1.3)	0.6 (0.0, 1.6)	0.0 (0.0, 1.0)	<0.001	0.0 (0.0, 1.3)	0.0 (0.0, 1.1)	0.12
Vegetables	0.2 (0.0, 0.5)	0.2 (0.0, 0.4)	0.2 (0.0, 0.4)	0.2 (0.0, 0.6)	0.31	0.2 (0.0, 0.4)	0.2 (0.0, 0.6)	0.17
Meat & proteinaceous foods	2.3 (1.6, 3.4)	3.2 (2.2, 4.1)	2.9 (2.1, 3.9)	2.7 (1.8, 4.0)	0.11	3.0 (2.1, 4.0)	2.7 (1.9, 4.0)	0.12
Extra foods	7.7(5.1, 10.3)	9.6 (6.4, 13.4)	7.8(5.7, 11.9)	7.7(5.4, 11.0)	0.036	8.9 (6.4, 12.7)	7.7 (5.4, 11.1)	0.024
**2-3 years**								
**Malaysia, n**	179	75	32	41		180	147	
Cereals & grains	2.2 (1.5, 3.0)	2.6 (1.9, 3.3)	2.7 (2.0, 3.4)	2.2 (1.6, 3.3)	0.071	2.2 (1.5, 3.0)	2.5 (1.8, 3.3)	0.023
Fruits	0.1 (0.0, 1.0)	0.3 (0.0, 0.9)	0.3 (0.0, 0.7)	0.0 (0.0, 0.8)	0.62	0.1 (0.0, 0.9)	0.3 (0.0, 0.9)	0.53
Vegetables	0.5 (0.1, 1.3)	0.5 (0.0, 1.5)	0.7 (0.0, 1.2)	0.0 (0.0, 0.5)	0.014	0.5 (0.1, 1.3)	0.4 (0.0, 1.2)	0.15

Meat & proteinaceous foods	0.9 (0.5, 1.4)	1.2 (0.7, 1.6)	1.1 (0.7, 1.6)	1.6 (1.0, 2.3)	<0.001	0.9 (0.5, 1.4)	1.3 (0.7, 1.9)	<0.001
Extra foods	2.0 (1.2, 3.4)	2.6 (1.3, 3.3)	4.1 (2.4, 5.5)	5.5 (2.8, 7.1)	<0.001	2.0 (1.2, 3.4)	3.1 (1.8, 5.6)	<0.001
**4-6 years**								
**Malaysia, n**	189	155	170	255		190	579	
Cereals & grains	3.0 (2.0, 3.7)	3.0 (2.2, 4.1)	3.2 (2.3, 4.3)	2.8 (2.0, 3.9)	0.039	0.1 (0.0, 0.9)	2.5 (1.8, 3.3)	0.98
Fruits	0.1 (0.0, 0.9)	0.3 (0.0, 1.0)	0.2 (0.0, 0.8)	0.0 (0.0, 1.0)	0.57	0.4 (0.0, 1.2)	0.3 (0.0, 0.9)	0.22
Vegetables	0.4 (0.0, 1.2)	0.4 (0.0, 1.5)	0.3 (0.0, 1.2)	0.2 (0.0, 1.0)	0.079	1.0 (0.5, 1.7)	0.4 (0.0, 1.2)	<0.001
Meat & proteinaceous foods	1.0 (0.5, 1.7)	1.3 (0.8, 2.1)	1.2 (0.7, 2.0)	1.3 (0.8, 1.8)	0.010	0.1 (0.0, 0.9)	1.3 (0.7, 1.9)	0.98
Extra foods	2.8 (1.5, 4.6)	2.9 (2.0, 4.8)	4.0 (2.1, 7.2)	4.7 (2.7, 7.6)	<0.001	2.8 (1.5, 4.6)	3.1 (1.8, 5.6)	<0.001
**7-12 years**								
**Malaysia, n**	49	193	336	774		53	1299	
Cereals & grains	4.0 (2.7, 5.2)	4.0 (3.1, 5.2)	3.8 (2.8, 5.0)	3.9 (2.8, 5.1)	0.24	4.0 (2.7, 5.2)	3.0 (2.1, 4.1)	0.87
Fruits	0.0 (0.0, 1.1)	0.3 (0.0, 1.1)	0.3 (0.0, 1.1)	0.0 (0.0, 0.8)	0.002	0.0 (0.0, 1.1)	0.1 (0.0, 1.0)	0.70
Vegetables	0.4 (0.0, 1.6)	1.1 (0.2, 2.5)	1.0 (0.1, 2.2)	0.6 (0.0, 1.6)	<0.001	0.4 (0.0, 1.6)	0.3 (0.0, 1.1)	0.23
Meat & proteinaceous foods	1.2 (0.7, 2.0)	1.6 (1.0, 2.4)	1.5 (0.9, 2.4)	1.6 (1.0, 2.5)	0.031	1.3 (0.7, 2.0)	1.3 (0.7, 2.0)	0.11
Extra foods	3.1 (1.9, 5.8)	4.5 (2.4, 7.5)	4.5 (2.5, 7.6)	5.2 (2.9, 8.4)	<0.001	3.1 (2.2, 6.0)	3.9 (2.3, 6.9)	0.003
**2-3 years**								
**Thailand, n**	376	170	96	82		376	348	
Cereals & grains	1.9 (1.1, 2.8)	2.1 (1.5, 3.1)	2.3 (1.7, 3.3)	2.3 (1.5, 3.4)	<0.001	1.9 (1.1, 2.8)	2.2 (1.6, 3.2)	<0.001
Fruits	0.2 (0.0, 1.2)	0.4 (0.0, 1.4)	0.5 (0.0, 1.3)	0.0 (0.0, 1.1)	0.21	0.2 (0.0, 1.2)	0.4 (0.0, 1.3)	0.35
Vegetables	0.3 (0.0, 0.8)	0.5 (0.0, 0.9)	0.3 (0.0, 0.8)	0.1 (0.0, 0.6)	0.027	0.3 (0.0, 0.8)	0.4 (0.0, 0.8)	0.24
Meat & proteinaceous foods	3.5 (1.9, 5.2)	3.7 (2.1, 5.2)	4.0 (2.2, 5.5)	4.3 (2.4, 6.0)	0.15	3.5 (1.9, 5.2)	3.9 (2.3, 5.4)	0.055
Extra foods	1.2 (0.6, 2.3)	1.4 (0.6, 2.4)	1.7 (0.9, 2.9)	2.7 (1.5, 3.6)	<0.001	1.2 (0.6, 2.3)	1.7 (0.8, 2.9)	<0.001
**4-6 years**								
**Thailand, n**	173	214	297	161		174	671	
Cereals & grains	2.7 (1.8, 3.5)	2.8 (1.9, 3.9)	2.7 (2.0, 3.7)	2.6 (1.8, 3.6)	0.29	2.7 (1.8, 3.5)	2.7 (1.9, 3.8)	0.11
Fruits	0.3 (0.0, 1.2)	0.3 (0.0, 1.1)	0.3 (0.0, 1.3)	0.0 (0.0, 1.3)	0.17	0.3 (0.0, 1.2)	0.3 (0.0, 1.2)	0.71
Vegetables	0.5 (0.0, 1.1)	0.5 (0.0, 1.1)	0.6 (0.0, 1.1)	0.2 (0.0, 0.9)	0.009	0.5 (0.0, 1.1)	0.5 (0.0, 1.1)	0.91
Meat & proteinaceous foods	4.0 (2.6, 5.7)	4.2 (2.9, 5.8)	4.1 (2.8, 6.2)	4.2 (2.6, 5.6)	0.64	4.0 (2.5, 5.7)	4.2 (2.8, 5.9)	0.30
Extra foods	1.7 (0.8, 3.4)	2.3 (1.1, 3.7)	2.8 (1.5, 4.5)	3.1 (1.6, 4.8)	<0.001	1.7 (0.8, 3.4)	2.6 (1.4, 4.3)	<0.001
**7-12 years**								
**Thailand, n**	70	143	403	461		70	1007	
Cereals & grains	3.0 (2.4, 4.5)	3.7 (2.5, 5.0)	3.5 (2.7, 5.0)	3.8 (2.7, 4.8)	0.13	3.0 (2.4, 4.5)	3.7 (2.7, 4.9)	0.022
Fruits	0.4 (0.0, 1.5)	0.1 (0.0, 1.5)	0.3 (0.0, 1.4)	0.0 (0.0, 0.9)	<0.001	0.4 (0.0, 1.5)	0.0 (0.0, 1.1)	0.16

Vegetables	0.8 (0.3, 1.4)	0.6 (0.1, 1.6)	0.9 (0.3, 2.1)	0.5 (0.0, 1.4)	<0.001	0.8 (0.3, 1.4)	0.8 (0.1, 1.7)	0.97
Meat & proteinaceous foods	4.8 (3.0, 6.7)	4.9 (3.2, 7.6)	4.3 (3.0, 6.3)	4.5 (2.9, 6.6)	0.072	4.8 (3.0, 6.7)	4.5 (3.0, 6.6)	0.81
Extra foods	3.5 (1.7, 5.5)	2.7 (1.5, 4.5)	3.3 (1.9, 5.0)	3.5 (2.0, 5.5)	0.029	3.5 (1.7, 5.5)	3.4 (1.9, 5.1)	0.75
**2-3 years**								
**Vietnam, n**	273	151	155	109		137	551	
Cereals & grains	4.2 (3.1, 5.6)	4.3 (3.5, 5.3)	4.2 (3.0, 5.6)	5.2 (4.0, 6.2)	<0.001	4.4 (3.1, 5.6)	4.4 (3.4, 5.7)	0.40
Fruits	0.3 (0.0, 1.0)	0.3 (0.0, 1.1)	0.3 (0.0, 1.1)	0.0 (0.0, 1.1)	0.083	0.3 (0.0, 1.1)	0.3 (0.0, 1.1)	0.80
Vegetables	0.4 (0.1, 0.6)	0.4 (0.2, 0.7)	0.4 (0.1, 0.8)	0.5 (0.2, 1.0)	0.049	0.3 (0.1, 0.6)	0.4 (0.2, 0.8)	0.032
Meat & proteinaceous foods	1.8 (1.1, 2.6)	1.9 (1.3, 2.6)	1.7 (1.1, 2.2)	1.7 (1.1, 2.4)	0.10	1.8 (1.0, 2.7)	1.7 (1.2, 2.5)	0.98
Extra foods	0.8 (0.3, 1.8)	1.3 (0.5, 2.4)	1.3 (0.4, 2.3)	1.4 (0.9, 2.5)	<0.001	0.8 (0.3, 1.8)	1.2 (0.5, 2.2)	0.007
**4-6 years**								
**Vietnam, n**	150	107	227	276		60	700	
Cereals & grains	5.8 (4.3, 7.2)	5.6 (3.9, 7.0)	5.6 (4.7, 7.1)	5.7 (4.4, 7.2)	0.54	6.1 (4.0, 7.3)	5.7 (4.4, 7.1)	0.59
Fruits	0.3 (0.0, 1.3)	0.5 (0.0, 1.3)	0.4 (0.0, 1.2)	0.0 (0.0, 1.0)	0.014	0.3 (0.0, 0.9)	0.2 (0.0, 1.2)	0.89
Vegetables	0.5 (0.1, 0.8)	0.4 (0.1, 0.8)	0.5 (0.2, 0.8)	0.4 (0.0, 0.8)	0.25	0.6 (0.0, 0.8)	0.4 (0.1, 0.8)	0.71
Meat & proteinaceous foods	2.7 (1.7, 3.8)	2.4 (1.6, 3.6)	2.4 (1.6, 3.3)	2.2 (1.5, 3.4)	0.12	2.5 (1.6, 3.6)	2.4 (1.6, 3.5)	0.58
Extra foods	1.2 (0.3, 2.2)	1.2 (0.5, 1.8)	1.4 (0.5, 2.5)	1.3 (0.5, 2.5)	0.13	1.0 (0.3, 2.2)	1.3 (0.5, 2.4)	0.20
**7-12 years**								
**Vietnam, n**	133	99	395	914		42	1499	
Cereals & grains	6.6 (4.9, 8.8)	6.0 (4.6, 7.8)	6.5 (5.4, 8.3)	6.7 (5.2, 8.7)	0.072	6.5 (4.9, 9.2)	6.6 (5.2, 8.5)	0.75
Fruits	0.1 (0.0, 1.3)	0.3 (0.0, 1.8)	0.4 (0.0, 1.5)	0.2 (0.0, 1.5)	0.69	0.3 (0.0, 1.9)	0.2 (0.0, 1.5)	0.98
Vegetables	0.5 (0.1, 1.1)	0.7 (0.1, 1.3)	0.5 (0.2, 1.1)	0.5 (0.1, 1.1)	0.65	0.3 (0.0, 1.3)	0.5 (0.1, 1.1)	0.17
Meat & proteinaceous foods	3.1 (2.2, 4.6)	3.3 (2.2, 4.9)	3.3 (2.1, 4.5)	3.0 (2.0, 4.3)	0.046	3.3 (2.2, 4.7)	3.1 (2.1, 4.4)	0.38
Extra foods	0.8 (0.0, 1.9)	1.0 (0.0, 2.5)	1.3 (0.6, 2.7)	1.4 (0.4, 2.8)	<0.001	1.0 (0.0, 3.2)	1.3 (0.4, 2.7)	0.56

Data shown as median interquartile range. Data were analyzed using Kruskal-Wallis tests with post hoc tests (corrected for number of tests). Differences between medians are indicated by different letters
